# A tumor suppressor protein encoded by circKEAP1 inhibits osteosarcoma cell stemness and metastasis by promoting vimentin proteasome degradation and activating anti-tumor immunity

**DOI:** 10.1186/s13046-024-02971-7

**Published:** 2024-02-21

**Authors:** Ying Zhang, Zhaoyong Liu, Zhigang Zhong, Yanchen Ji, Huancheng Guo, Weidong Wang, Chuangzhen Chen

**Affiliations:** 1 Department of Radiotherapy, Cancer Hospital of Shantou University Medical College, No. 7 Raoping Road, Shantou, Guangdong 515041 PR China; 2 Department of Orthopaedics, First Affiliated Hospital of Shantou University Medical College, No. 57 Changping Road, Shantou, Guangdong 515041 China; 3 Sports Medicine Center, First Affiliated Hospital of Shantou University Medical College, Shantou, 515041 China; 4 Sports Medicine Institute, Shantou University Medical College, Shantou, 515041 China; 5 Department of Orthopaedics, Cancer Hospital of Shantou University Medical College, No. 7 Raoping Road, Shantou, Guangdong 515041 China

**Keywords:** circKEAP1, Osteosarcoma, Vimentin, ARIH1

## Abstract

**Background:**

Osteosarcoma (OS) is one of most commonly diagnosed bone cancer. Circular RNAs (circRNAs) are a class of highly stable non-coding RNA, the majority of which have not been characterized functionally. The underlying function and molecular mechanisms of circRNAs in OS have not been fully demonstrated.

**Method:**

Microarray analysis was performed to identify circRNAs that are differentially-expressed between OS and corresponding normal tissues. The biological function of circKEAP1 was confirmed in vitro and in vivo. Mass spectrometry and western blot assays were used to identify the circKEAP1-encoded protein KEAP1-259aa. The molecular mechanism of circKEAP1 was investigated by RNA sequencing and RNA immunoprecipitation analyses.

**Results:**

Here, we identified a tumor suppressor circKEAP1, originating from the back-splicing of exon2 of the *KEAP1* gene. Clinically, circKEAP1 is downregulated in OS tumors and associated with better survival in cancer patients. N6-methyladenosine (m6A) at a specific adenosine leads to low expression of circKEAP1. Further analysis revealed that circKEAP1 contained a 777 nt long ORF and encoded a truncated protein KEAP1-259aa that reduces cell proliferation, invasion and tumorsphere formation of OS cells. Mechanistically, KEAP1-259aa bound to vimentin in the cytoplasm to promote vimentin proteasome degradation by interacting with the E3 ligase ARIH1. Moreover, circKEAP1 interacted with RIG-I to activate anti-tumor immunity via the IFN-γ pathway.

**Conclusion:**

Taken together, our findings characterize a tumor suppressor circKEAP1 as a key tumor suppressor regulating of OS cell stemness, proliferation and migration, providing potential therapeutic targets for treatment of OS.

**Supplementary Information:**

The online version contains supplementary material available at 10.1186/s13046-024-02971-7.

## Introduction

Osteosarcoma (OS) is the most common primary bone tumor of adolescents, and both frequently metastasizes to the lungs and is resistant to chemoradiotherapy, resulting in poor prognosis [1]. Aberrant molecular signaling pathways and non-coding (nc) RNAs can provide valuable insight into mechanisms underlying the development and stemness of OS, as well as provide biomarkers to explore mechanism-based therapies to improve OS patient survival [2].

A key ncRNA subfamily, denoted circular RNAs (circRNAs) is generated by back splicing of mRNA, and lacks the 5’-3’ polarity and a poly-A tail of mRNAs [3]. Recent studies have demonstrated that circRNA plays important roles in the physiology and pathology of cells [4]. High-throughput sequencing discovered that amount of circRNAs are abnormally expressed and exert function in various cancers, including OS [4, 5]. For instance, circTADA2A is upregulated in both OS tissues and cell lines [6]. CircECE1 mediates the Warburg effect through the c-Myc/TXNIP axis in OS [7]. CircMYO10 promotes chromatin remodeling via the miR-370-3p/RUVBL1 axis and increases transcriptional activity of the β-catenin/LEF1 complex to promote OS progression [8]. However, the knowledge that circRNAs could regulate the tumorigenesis and stemness of OS is only at the preliminary level and need to be further explored.

N6-methyladenosine (m6A), the most common post-transcriptional RNA modification, regulates RNA transcription, splicing, degradation, and translation [9]. RNA m6A modification of RNA is performed by m6A methyltransferases (writers), such as methyltransferase-like 3 proteins (METTL3) and METTL14 as well as m6A demethylases (erasers), such as fat mass and obesity-associated protein (FTO) and human AlkB homolog H5 (ALKBH5), whereas readers YT521-B homology domain (YTHDF1/2) mediate m6A-dependent functions [10]. CircRNAs modified by m6A can be affected by writers, erasers, and readers, which modulate the translation and degradation of circRNAs, resulting in altered expression [11]. Moreover, dysregulation of m6A modification has been linked to initiation, metastasis, drug resistance, and other OS processes [12]. However, how m6A modification mediates circRNA expression or function need to be explored.

Retinoic acid-inducible gene-I (RIG-I) is a RIG-I-likereceptor (RLR) family member that functions as a pathogen recognition receptor (PRR) to trigger the innate immune response against viral infections [13]. Recent studies have demonstrated that RIG-1 expression can be increased by virus infection, LPS and interferons (IFN) [14]. RIG-I signaling activates transcriptional activation and expression of pro-inflammatory genes, including type I IFNs and its downstream genes, such as signal transducers and activators of transcription (STATs) and interferon regulatory factors (IRFs) [15]. RIG-I has been reported to serve as a tumor suppressor by promoting innate immune activation and immunogenic cell death in hepatocellular carcinoma (HCC) and cervical cancer [16]. The underlying mechanisms by which RIG-I increases infiltration of immune cells and enhances anti-cancer effects and reduces immunosuppressive activities remain to be defined.

Here, we identify a circular RNA, circKEAP1, derived from the tumor suppressor gene Kelch-like ECH-associated protein 1 (KEAP1). We show that m6A lowers circKEAP1 expression in OS. Overexpression of circKEAP1 inhibits OS cell proliferation, migration and stemness. Mechanistically, circKEAP1 contains a large open reading frame (ORF) and encodes a truncated protein, KEAP1-259aa, that suppresses OS malignancy by interacting with E3 ligase Ariadne-1 homolog (ARIH1) to promote vimentin degradation. We further show circKEAP1 activates anti-tumor immunity through the RIG-I signaling pathway, providing important insight into the immune response in OS.

## Materials and methods

### Patient samples and cell lines

OS tissues and normal adjacent tissues from patients with primary OS who underwent radical resection were collected from both the First Affiliated Hospital of Shantou University Medical College and Cancer Hospital of Shantou University Medical College from 2015 to 2021.In this cohort, 48 (66.7%) were men and 24 (33.3%) were women; the age was 8–69 years, with a median of 25 years. Twenty-one cases (29.1%) were located in the femur, whereas 26 (36.1%) cases were located in the jawbone, three cases located in the tibia and the remaining were located in the other part of body. A total of 12.5% of patients (*n* = 9) showed lung metastasis. Tumor invasion was found in 12.5% of the tumors (*n* = 9). All participants involved in our study provided written informed consents. This study was approved by the ethical review committees of the Cancer Hospital of Shantou University Medical College.

HEK293T human embryonic kidney cells, the hFOB1.19 osteoblast cell line, and OS cell lines U2OS, Saos-2, HOS and MG63 were purchased from the Type Culture Collection of the Chinese Academy of Sciences (Shanghai, China), and maintained in Dulbecco’s modified Eagle’s medium (DMEM) or Roswell Park Memorial Institute (RPMI) 1640 (Gibco, Waltham, MA, USA) supplemented with 10% fetal bovine serum (Gibco) and 1× antibiotic mixture (Yeasen, Shanghai, China). hFOB1.19 was cultured in medium (DMEM/F12 supplemented with 0.3 mg/ml G418, 10% FBS and 1% P/S) purchased from Procell (Wuhan, China), maintained at 33.5 °C in a humidified atmosphere of 5% CO_2_. Cell lines were confirmed to be mycoplasma negative and authenticated by short tandem repeats (STR) profiling.

### Human circRNA microarray analysis

Total RNA from OS sample and case matched normal tissues was isolated using TRIzol (Invitrogen, MD, USA) and quantified using the NanoDrop ND-1000. The sample preparation and microarray hybridization were performed based on standard Arraystar’s protocols (Aksomics, Shanghai, China). Briefly, total RNA was digested with RNase R (Epicentre, Inc.) to remove linear RNAs and enrich circular RNAs. Then, the enriched circular RNAs were amplified and transcribed into fluorescent cRNA utilizing a random priming method (Arraystar Super RNA Labeling Kit; Arraystar). The labeled cRNAs were hybridized onto the Arraystar Human circRNA Array V2 (Arraystar). After washing the slides, the arrays were scanned by the Agilent Scanner G2505C.

### Stable cell lines and plasmids transfection

Human circKEAP1 overexpression vectors, KEAP1-259aa overexpression vectors, IRSE mutant overexpression vectors, ATG mutant overexpression vectors, vimentin overexpression vectors, short hairpin(sh) RNA vectors targeting circKEAP1 and empty lentivirus vector were purchased from the Vigene Company (Shandong, China). After lentiviral vectors were co-transfected with packaging plasmids psPAX2 and pMD2.G into HEK293T cells, supernatant was harvested at 48 and 72 h and used for infection of the OS cells. After 48 h, stably infected cell lines were obtained by selection with puromycin as per the manufacturer’s suggested protocol.

Full length circKEAP1, start codon ATG-mutated circKEAP1, IRSE-mutant circKEAP1 KEAP-259aa and control vector were cloned into a CMV promoter-dependent flag-tagged expression vector (Vigene). GFP-tagged wild-type or mutant vimentin, Myc-tagged ARIH1, and HA-tagged wild-type or mutant ubiquitin vectors were purchased from Vigene. Transfection was carried out using jetPRIME (Polyplus Transfection, Guangzhou, China) according to the manufacturer’s instructions.

### Drugs and siRNAs

RNase R (20 U/µl, Yeasen) was used to treat total RNA (20 µg) at 37 °C for 15 min. DNA transcription and replication inhibitor actinomycin D (Act D, HY-17,559), protein synthesis inhibitor cycloheximide (CHX, HY-12,320), and vimentin inhibitor Withaferin A (WFA, HY-N2065) were purchased from MedChemExpress (Shanghai, China). SiRNAs targeting ARIH1, vimentin, FTO, METTL3, and METTL14 were purchased from GenePharma (Jiangsu, China).

### Western blot and liquid chromatography-mass spectrometry (LC-MS)/MS

Total cell extracts were lysed in RIPA buffer containing protease and phosphatase inhibitor cocktail (Beyotime, Shanghai, China). Western blot was performed as previously described [17]. Primary antibodies against KEAP1 (1:500, sc-514,914, USA) and ARIH1 (1:500, sc-514,551) were purchased from Santa Cruz (MD, USA). Primary antibodies against Cleaved-caspase-3 (#9664), caspase-3 (#9662), PARP (#9542), cleaved-PARP (#9541), vimentin (#5741), ubiquitin (#8240), Flag (#14,793), HA(#3724), GFP (#2555), FTO (#31,687), METTL3 (#86,132), METTL14 (#51,104), YTHDF2 (#80,014), STAT1 (#9172),p-STAT1 (#8826), P65 (#8242), p-p65 (#3033), RIG-I (#3743), IRF7 (#4920), IRF3 (#11,904), p-IRF7 (#24,129), p-IRF3 (#4947), PCNA (#2586), SOX2 (#23,064), ABCG2 (#4477), NAONG (#4903), GAPDH (#2118), and β-actin (#4967) were purchased from Cell Signaling Technology. Densitometry analysis of Western blots was performed using Image J and the band densities were normalized to GAPDH. The control was set to 1 for plotted graphs. Protein identification was performed by LC-MS/MS. Briefly, 50 µg proteins was subjected to SDS-PAGE and the silver stained protein bands were excised and analyzed by LC-MS/MS (BGI, Shenzhen, China.

### RNA extraction, PCR and quantitative RT-PCR

Total RNA was extracted with a Cell/Tissue RNA Isolation kit (Vazyme, Nanjing, China) according to the manufacturer’s instructions. HiScript II RT SuperMix (Vazyme) was used to synthesize first-strand cDNA. QRT-PCR was performed with ChamQ SYBR qPCR (Vazyme) using a CFX96 QPCR Detection System (BIO-RAD, CA, USA). The running conditions for qPCR were as follows: for activating the DNA polymerase, hot start was performed for 30 s at 95 °C, and then cycling at 95 °C for 10 s and 60 °C for 30 s for a total of 40 cycles. PCR was subsequently performed by 2×Taq PCR Mix kit (Absin, Shanghai, China), and the product was run on a 2% agarose gel. The running conditions for PCR were as follows: for activating the DNA polymerase, hot start was performed for 2 min at 94 °C, and then cycling was done at 94 °C for 30 s, 60 °C for 30 s and 72 °C for 60 s for a total of 30 cycles. The primer sequences are listed in Supplemental Table 1.

### Non-adherent sphere formation assay

After transfection, single-cell suspensions were seeded in 6-well non-adherent plates at a density of 2,000 cells per well. The sphere medium contained phenol red-free DMEM/F-12 (Invitrogen), 2% B27 supplement (Thermo Fisher Scientific), 4 U/L insulin (Beyotime), 20 ng/ml basic fibroblast growth factor (bFGF, BD Biosciences, CA, USA) and 20 ng/ml epidermal growth factor (EGF, BD Biosciences). After 72 h, the number of sarcospheres was counted under the microscope and the image was captured with a microscope (Leica, Mannheim, Germany).

### Flow cytometry

Human CD133 Alexa Fluor 700-conjugated antibody was obtained from RD Systems (MN, USA). OS cells were incubated with CD133 antibody in PBS for 15 min and then analyzed by flow cytometry (C6, Japan). For apoptosis, an Annexin V- Apoptosis Detection kit (Bioss, Beijing, China) was used. A mix of Annexin V-AF647 and PI staining allows for distinction between early (Annexin+/PI-) and late (Annexin+/PI+) apoptotic cells, necrotic cells and live cells. For cell death quantitation, 1 × 10^5^ cells were collected and washed with pre-cooled PBS. After adding binding buffer with 5 µl Annexin V-AF647 and 10 µl PI staining solution, cells were incubated at room temperature for 10–15 min, then flow cytometry was performed (C6, Japan). The proportion of apoptotic cells (Annexin-V positive cells) were shown as the mean ± SD.

### Immunohistochemistry (IHC) and in situ hybridization (ISH)

For IHC, all tissues were deparaffinized and hydrated, and then heated by microwaving in 0.01 mol/L sodium citrate buffer (pH = 6.0) for 15 min. After treating with 0.3% hydrogen peroxide and blocking with 5% BSA for 30 min, specimens were incubated with Ki67 antibody (#12,202, CST, 1:200), or CD8 antibody (sc-1181, Santa cruz, 1:500) overnight followed by washing and incubating with biotinylated anti-rabbit IgG for 1.5 h. Sections were counterstained with 0.1% hematoxylin. For ISH, the tissues were hybridized with a specific digoxin-labeled circKEAP1 probe (Geneseed, Guangzhou, China) according to the manufacturer’s instructions. ISH was performed using an ISH kit (Boster) based on the manufacturer’s guidelines. For control, the enclosed negative control sense probe was applied. Expression of Ki67 or circKEAP1 was quantified and analyzed from 5 randomly selected fields of view by two pathologists. Proportion percentages were scored as follows: 0, < 10%; 1, 10–25%; 2, 26–50% ; 3, 51–75% and 4, > 75%.Staining intensity was scored as follows: 0, no staining; 1, weak; 2, moderate; 3, strong. The final score was the proportion score multiplied by the intensity score and the cutoff score for high or low expression was 6.

### Human inflammation antibody array

For semi-quantitative detection of 40 human proteins in cell culture media, we used a Human Inflammation Antibody Array (AAH-INF-3-8, RayBiotech Company, Norcross, GA). After blocking the membrane for 30 min at room temperature, we pipetted the media sample and incubated overnight at 4 °C. After washing, a biotinylated antibody cocktail and HRP-streptavidin was added into each well and membranes were incubated for 2 h. Then membranes were exposed to the chemiluminescence imaging system.

### ELISA

Transfected cell culture supernatants were collected and analyzed using ELISAs according to the manufacturer’s instructions. ELISA kits for human CXCL10 and IFNγ were purchased from Absin, (Shanghai, China). ELISA kits for human CCL5 were purchased from Sino Biological (USA).

### Methylated RNA immunoprecipitation (MeRIP)

M6A modifications on circKEAP1 were determined using the MeRIP m6A Kit (BersinBio, Shanghai, China) according to the manufacturer’s instructions. Briefly, 300 nt length RNAs sheared by ultrasonication were incubated with m6A antibody or IgG-conjugated beads at 4 °C overnight. RNA –antibody complex was incubated with protein A/G magnetic beads at 4 °C for 3 h and then eluted with elution buffer. After extracting the RNA, PCR or qRT-PCR was performed and calculated by normalizing to input.

### RNA immunoprecipitation (RIP) and biotin RNA pulldown assay

RIP was performed using a RIP kit (BersinBio) according to the manufacturer’s instructions. Briefly, Protein A/G agarose beads were coated with 5 µg antibodies against FTO (CST), METTL3 (CST), and m6A (Abcam) or IgG (Thermo Fisher Scientific). Then the beads-antibody complexes were incubated with cell lysates overnight at 4 °C, washed and then subjected to elution buffer and proteinase K digestion. Finally, the RNA was extracted and determined by qPCR or PCR, and normalized to input. For RNA pull-down assays, an RNA pulldown kit (BersinBio) was used according to the manufacturer’s instructions. Briefly, 1 × 10^7^ cells were lysed and incubated with 3 µg of biotinylated oligo probes against endogenous circKEAP1 (Exon Biotechnology, Guangzhou, China) at 4 °C overnight. Then streptavidin-coated magnetic beads were added and lysates were further incubated at room temperature for 2 h. After washing, western blotting was performed to detect the pulled-down proteins.

### Co-IP

After adding IP lysis buffer (Beyotime) in OS cells, the supernatants were collected and incubated with 4 µl of anti-vimentin (CST) or anti-Flag antibodies (CST) at 4 °C overnight. Then 40 µl of Protein A + G Magnetic Beads (Beyotime) were added and the mixture was incubated at room temperature for 2 h. After washing with PBS, immunoprecipitates beads were eluted with SDS-loading buffer at 98 °C for 5 min and the proteins were analyzed by western blot analysis.

### Luciferase reporter assay

Wild-type or mutant IRES sequences in circKEAP1 were amplified and subcloned into the pGL3 vector (Vigene). For the luciferase assays, cells were plated in 12-well plates, then the reporter plasmid and pRL-TK were co- transfected into cells using JetPRIME (Polyplus Transfection). After 48 h, a Dual-Luciferase Reporter assay kit (Promega, MD, USA) was used to measure luciferase activity and the results were expressed the ratio of from firefly to Renilla luciferase luminescence ratio.

### Cell counting Kit-8 (CCK8) and cell migration assay

OS cells were transfected with the indicated plasmids. After 48 h, 1 × 10^3^ cells were placed in each well of 96-well microplate for 4 days. Then cells were incubated with 10 µl CCK8 reagent (Liji, Shanghai, China) for 2 h, and absorbance at 450 nm relative to a blank well was measured. For cell migration, transwell and wound healing assays were performed as described previously [17]. After transfection, 2 × 10^5^ OS cells were cultured in the serum-free medium for 12–16 h and then suspended in serum-free medium. The cells were seeded into the upper chambers and 500 µl complete medium was added into the bottom chambers (Corning, NY, USA) for migration assays. After 24 h, the cells on the lower compartment were fixed in 4% paraformaldehyde (Beyotime) and stained with crystal violet (Solarbio), then photographed and counted with an optical microscope (Olympus, Tokyo, Japan). For the wound healing assay, cells were seeded in 6-well plates at a density of 1 × 10^6^ cells per well. a straight scratch was made using a 200 µl pipette tip when the density reached approximately 100%. After washing with PBS, the loose cells were removed and cells were cultured with serum-free medium. Images were taken with an optical microscope (Olympus) each day for 3 days. Image J software was used to measure the relative wound areas.

### Fluorescence in situ hybridization (FISH) and immunofluorescence (IF) staining

The FISH assay was performed in OS cells using fluorescence in situ hybridization kit (RiboBio) according to the manufacturer’s manual. Biotin-labeled probes specific for circKEAP1 probes were purchased from BOSTER (Wuhan, China). FISH-IF staining was performed preformed as described previously [18]. Briefly, cells were seeded and grown onto coated cover slips for 24 h. After fixing with 4% paraformaldehyde and digestion with proteinase K, the slides were incubated with pre-hybridization solution at 37 °C for 1 h and then the probe hybridization was allowed to proceed at 37 °C overnight. Slides were washed in SSC solution and incubated with primary antibodies overnight. After washing, cells were incubated with appropriate secondary antibodies (Absin) at room temperature for 1 h. Then nuclei were counterstained with DAPI after washing. Images were captured with a Zeiss confocal microscope (Oberkochen, Germany).

### Animal studies

Stable circKEAP1-overexpression, KEAP1-259aa-overexpression, IRSE mutant-overexpression, ATG mutant-overexpression, vimentin-overexpression, circKEAP1+-vimentin-overexpression or control OS cells (1 × 10^6^) were suspended in Matrigel matrix (1:1, Corning, NY, USA) and subcutaneously injected into the flank of 5-week-old male BALB/c nude mice (*n* = 4–6 per group). Tumor size was measured every 7 days for a total period of 4 weeks. Tumor volume was determined using the formula: volume = length×width^2^/2. To evaluate metastasis, Stable circKEAP1-overexpression, KEAP1-259aa-overexpression, IRSE mutant-overexpression, ATG mutant-overexpression, control OS cells (5 × 10^5^) were injected into mice through the tail vein (*n* = 6 per group). After five weeks, euthanasia was administered and the numbers of metastatic nodules of the lungs were counted under the microscope. All procedures were approved by the Institutional Animal Care and Use Committee of Shantou University Medical College.

### Statistical analysis

Experiments were performed three times independently and the data are expressed as mean ± SD. The data was analyzed using SPSS software version 20.0 (SPSS Inc., Chicago, IL) and GraphPad 8.0. The survival analysis was measured by Kaplan-Meier curve. Student’ t-test and one-way ANOVA were used to assess differences in variables between groups. The correlation between different expressions was tested by the Pearson correlation coefficient. Differences were statistically significant when *P* < 0.05.

## Results

### Identification of circKEAP1 and its clinical significance in OS tissues

**Fig. 1:**
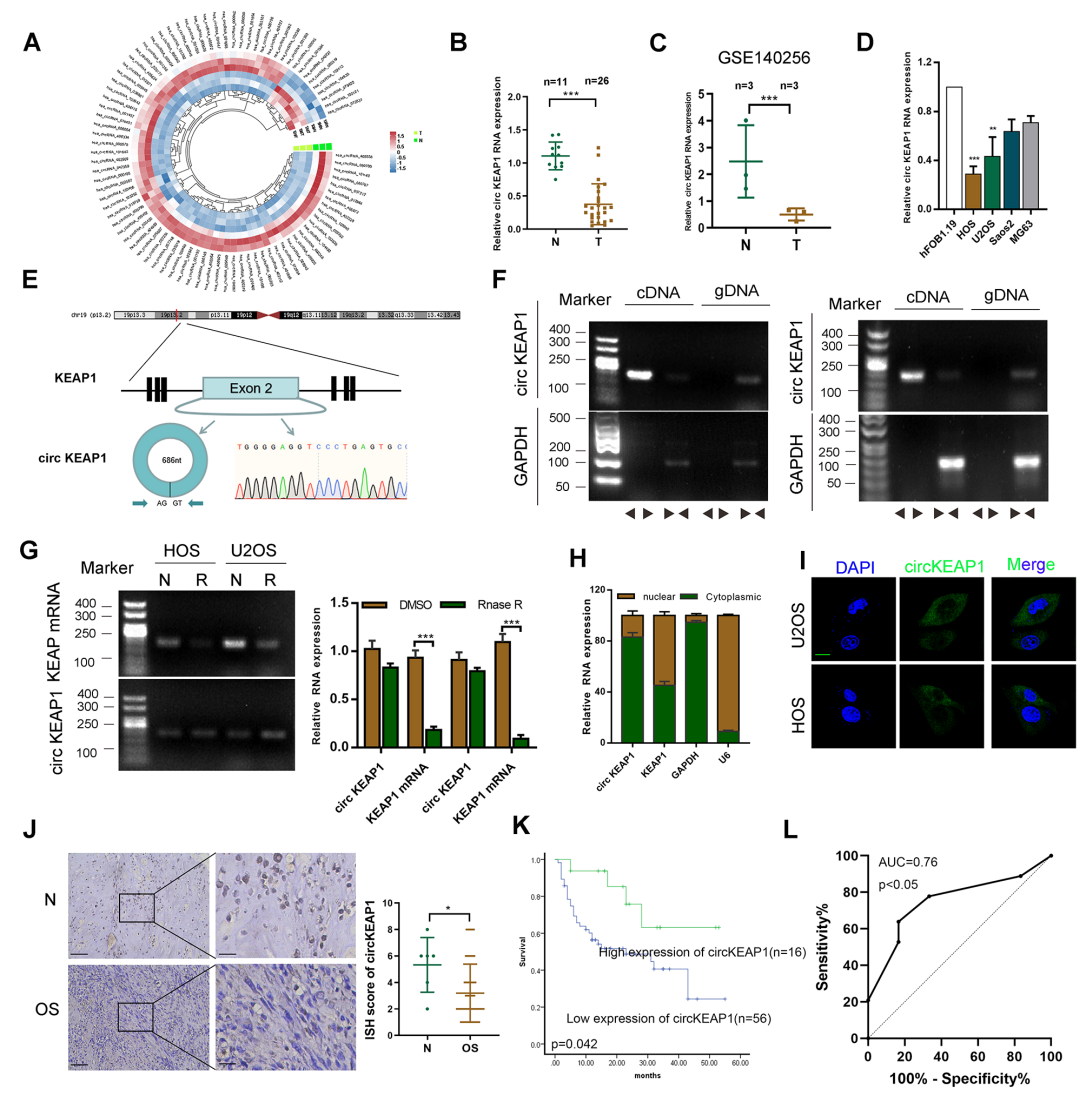
Validation and expression of circKEAP1 in OS tissues. (**A**). A circRNA microarray was used for 3 pairs of OS tissues and case-matched normal tissues. Circular heatmap showing the differentally-expressed circRNAs. (**B**). Expression of circKEAP1 was detected by qRT-PCR in OS (*n* = 26) and normal tissues (*n* = 11) from our own tissue bank. (**C**). The expression of circKEAP1 in normal (*n* = 3) and OS tissues (*n* = 3) was also found in GSE140256 from GEO database. (**D**). CircKEAP1 expression was detected by qRT-PCR in various human OS cell lines (HOS, Saos-2, MG63 and U2OS) and osteoblast cells (hFOB1.19). **, *** indicates significant differences compared with the hFOB1.19 at a *p* value < 0.01, < 0.001, respectively. (**E**). Schematic illustration showing the formation and head-to-tail splicing site of circKEAP1 via the circularization of exon 2 in KEAP1. The presence of circKEAP1 was validated by Sanger sequencing. (**F**). RT-PCR validated the existence of circKEAP1 in HOS and U2OS cell lines. CircKEAP1 was amplified by divergent and convergent primers in complementary DNA (cDNA) and genomic DNA (gDNA). (**G**). The expression of circKEAP1 and KEAP1 mRNA in both HOS and U2OS cell lines was detected by RT-PCR and qRT-PCR with/without RNase R (20 U/µl) treatment. (**H**). Expression of KEAP1 and circKEAP1 was detected in the cytoplasm and nuclei of OS cells. (**I**). FISH images demonstrating circKEAP1is mainly located in the cytoplasm of OS cells (scale bars, 20 μm). (**J**). Expression of circKEAP1 was detected by ISH in OS tissues (*n* = 72) and normal tissues (*n* = 6) (scale bars, 100 μm, 50 μm). (**K**). Overall survival analysis based on circKEAP1 expression in OS. (**L**). ROC curve was shown based on the expression of circKEAP1 in OS tissues. Error bars represent three independent experiments. *, **, *** indicates significant differences compared with the normal group at a *p* value < 0.05, < 0.01, < 0.001, respectively

To characterize differentially-expressed circular RNA transcripts, we conducted a microarray analysis on 3 pairs of OS and their corresponding normal tissues and identified a series of circRNAs (Fig. 1A). The Gene Ontology (GO) analysis of the different expressed circRNA is shown in Figure S1A. We then chose the top 5 most significantly differentially-expressed circRNAs and verified their expression levels in OS and normal tissues to confirm the microarray results (Figure S1B). Using divergent primers to specifically target the circular junction and sequencing validation, we found that circKEAP1 (hsa_circ_0049271) was one of the most clearly downregulated circRNAs in OS, as detected by RT-qPCR and lower in OS tissues than in normal tissues in our cohort (Fig. 1B). Similar results were found in the public GSE140256 dataset from GEO database (Fig. 1C). Also, we determined the expression of circKEAP1 in osteoblast cells hFOB1.19 and OS cells (Saos2, MG63, HOS and U2OS) and found the expression was higher in hFOB1.19 than OS cells (Fig. 1D).

CircKEAP1 has been mapped to exon 2 of the KEAP1 gene (686 bp). Sanger sequencing was performed to confirm the head-to-tail splicing of the amplified circKEAP1 (Fig. 1E). Using divergent and convergent primers, PCR results showed that circKEAP1 was amplified only from cDNA but not gDNA, indicating that the loop structure of circKEAP1 is generated from back-splicing (Fig. 1F). To further evaluate the stability and localization of circKEAP1, we treated the RNA with RNase R and found circKEAP1 was resistant to RNase R degradation compared with the linear form of KEAP1 mRNA, which suggested that circKEAP1 is highly stable (Fig. 1G). Subsequently, fluorescence in situ hybridization (FISH) and qRT-PCR following cell fractionation results showed that circKEAP1 was mainly localized in the cytoplasm rather than the nucleus (Fig. 1H, I). By ISH staining, we found that the expression level of circKEAP1 was lower in OS tissues than normal tissues (Fig. 1J). However, due to the limited number of OS tissues, we did not find a correlation between circKEAP1 expression and OS patient clinical parameters (Supplemental Table 2). Kaplan-Meier survival analysis found that OS patients who had higher levels of circKEAP1 had longer overall survival (Fig. 1K). Additionally, ROC curve analysis was performed and gave an AUC was 0.76 (95% CI: 0.5839–0.9231, *p* < 0.05, Fig. 1L), indicating that circKEAP1 has good sensitivity and specificity as a diagnostic marker for OS.

### CircKEAP1 inhibits the proliferation, migration and stemness of OS cells

**Fig. 2:**
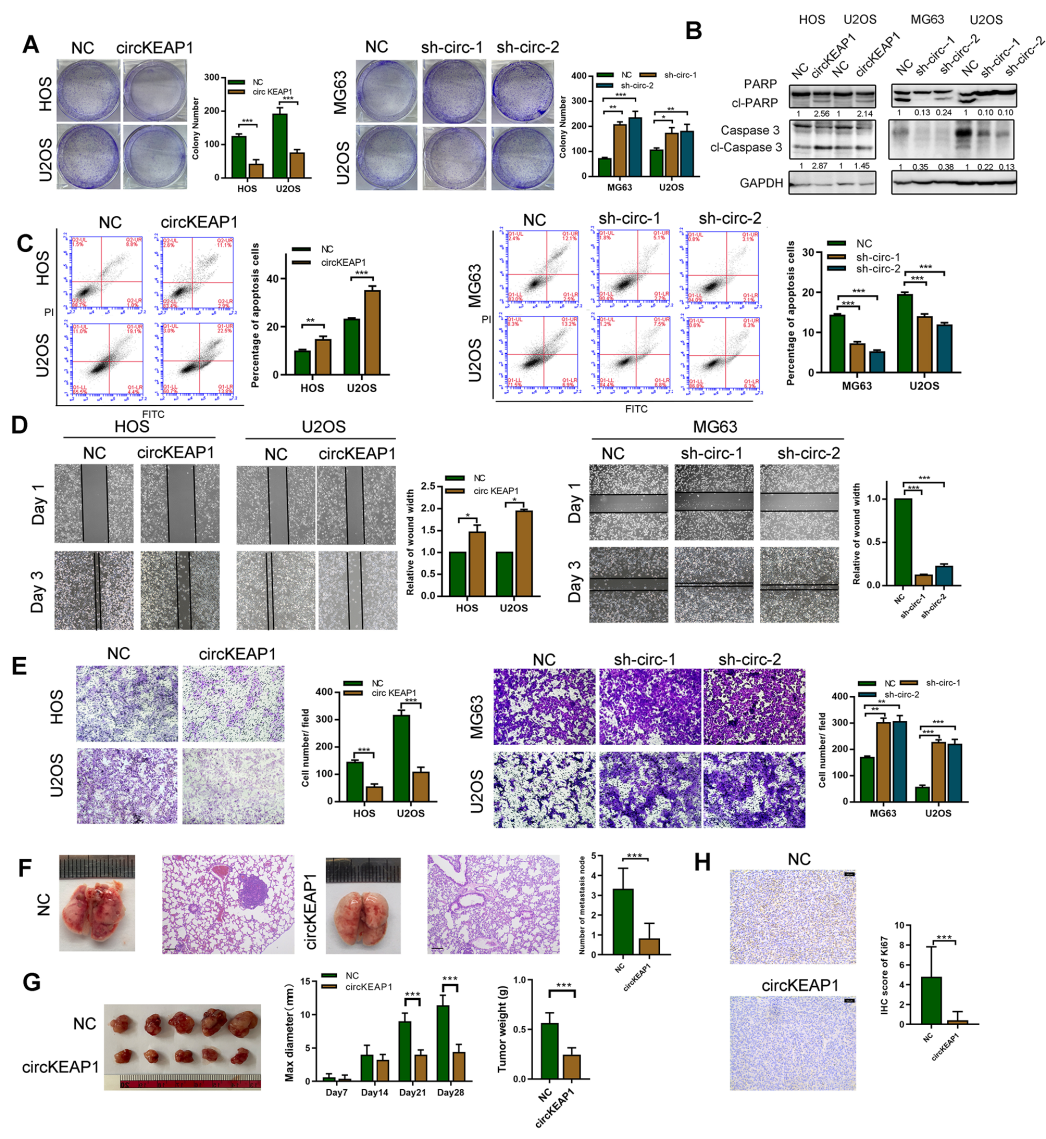
Tumor suppressive functions of circKEAP1 in vivo and in vitro. (**A**). OS cells were transfected with circKEAP1, two circKEAP1 shRNAs or control vectors. Colony formation of OS cells was determined in the indicated transfection groups. (**B**). Caspase 3 and PARP levels were determined by western blotting in the indicated transfection groups. (**C**). Apoptosis of the indicated OS cells was detected by flow cytometry. (**D**). Cell migration of the indicated OS cells was determined by wound healing assay. (**E**). Cell migration of indicated OS cells was determined by transwell assay. (**F**). The indicated U2OS cells (NC and circKEAP1) were injected into nude mice via tail vein and the lungs were harvested after 35 days. Representative image and H&E staining of mouse lung metastasis is shown (scale bars, 200 μm). (**G**). The indicated U2OS cells (NC and circKEAP1) were injected subcutaneously into nude mice (*n* = 5 per group) and tumor volume was measured every 7 days. After 28 days, tumors were collected and weighed. (**H**). Tumor tissues stained with Ki67 antibody and IHC score are shown (scale bars, 50 μm). Error bars represent three independent experiments. *, **, *** indicates significant differences compared with the control group at a *p* value < 0.05, < 0.01, < 0.001, respectively

To investigate the biological function of circKEAP1 in OS, stable circKEAP1-overexpressing HOS and U2OS cells were generated. Furthermore, shRNAs targeting the back-splice region of circKEAP1 were transfected into U2OS and MG63 cells. The transfection efficiency was confirmed by qRT-PCR (Figure S2A). Of note, shRNAs did not affect the mRNA level of KEAP1 (Figure S2B). We found that overexpression of circKEAP1 inhibited the proliferation and colony formation of OS cells as determined by CCK8 and colony formation assays (Fig. 2A and S2C). Subsequent western blotting and flow cytometry assays showed that apoptosis of OS cells was elevated by circKEAP1 (Fig. 2B, C). Similarly, the suppression of cell migration was also confirmed by transwell and wound healing assays (Fig. 2D, E). On the contrary, circKEAP1 shRNA increased colony formation proliferation and migration of OS cells (Fig. 2A-E). Cancer stem cells (CSCs) are a small subset of cancer cells, a primary cause of drug resistance, tumor relapse and metastasis of OS cells [19]. As shown in Figure S2D-F, stem cell markers, such as ABC2G, NANOG, SOX2, and CD133, were downregulated in circKEAP1-transduced U2OS and HOS cells detected by western blotting, qRT-PCR and immunofluorescence. Flow cytometry indicated that the proportion of CD133-positive cells was decreased after the circKEAP1 was overexpressed in U2OS and HOS cells (Figure S2G). Sarcosphere assays showed that circKEAP1-overexpressing cell lines formed significantly smaller and fewer sarcospheres (Figure S2H). Collectively, these results suggested that circKEAP1 inhibited the proliferation, migration and stemness of OS cells.

To further confirm our *in-vitro* findings, we verified the biological role of circKEAP1 in vivo using a xenograft model. U2OS cells stably expressing circKEAP1 were subcutaneously implanted into nude mice. In vivo metastatic assays showed that circKEAP1 decreased detectable lung metastasis compared with the control group (Fig. 2F). Consistent with in vitro findings, circKEAP1 dramatically attenuated tumor growth in vivo (Fig. 2G). We then performed immunohistochemistry for Ki67, a marker of proliferating cells. Weaker Ki67 staining was found in circKEAP1-overexpressing tumors compared to the control group (Fig. 2H).

### CircKEAP1 encodes a 259-amino acid (aa) novel protein KEAP1-259aa

**Fig. 3:**
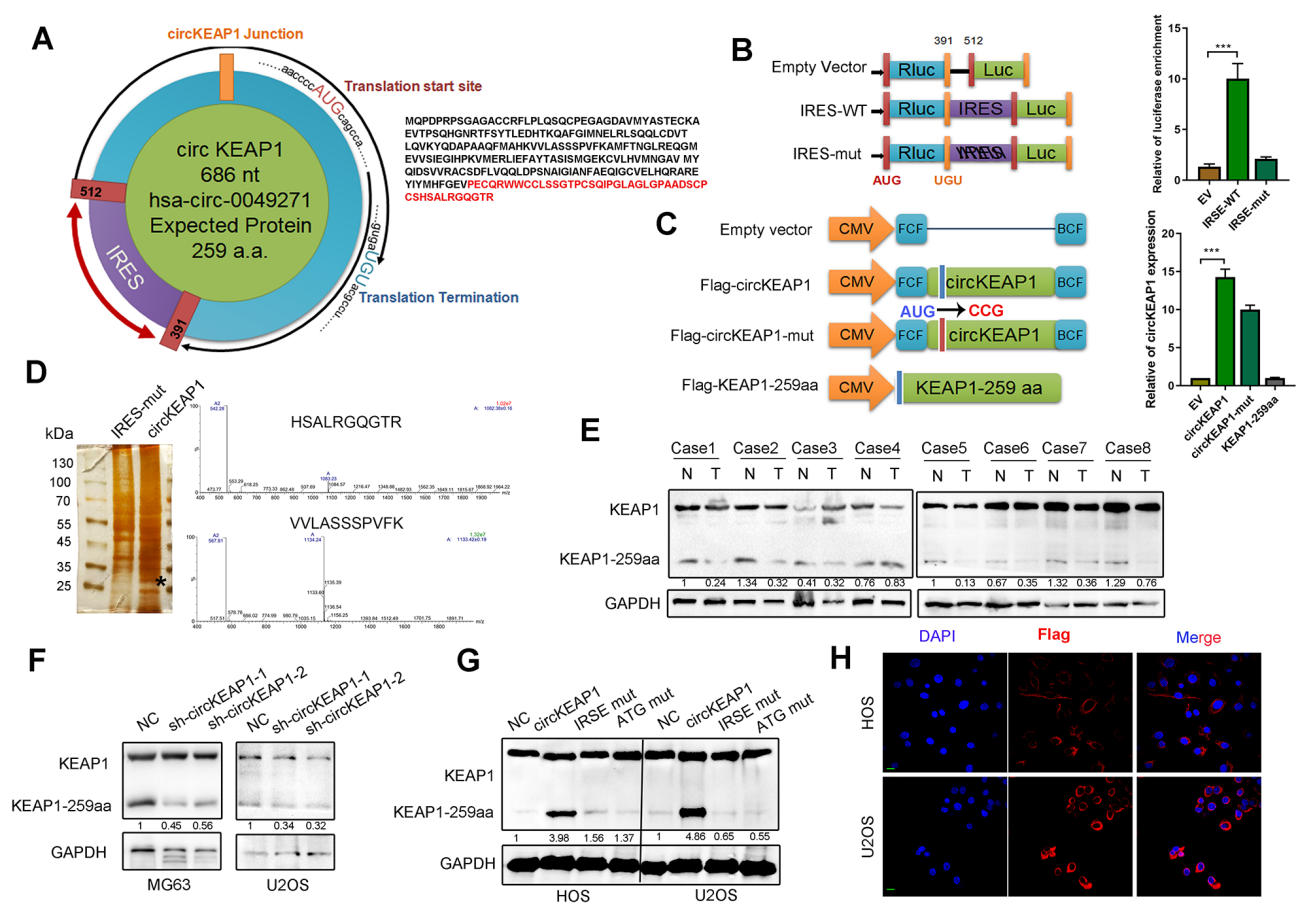
CircKEAP1 encodes a novel 259-amino acid protein denoted KEAP1-259aa. (**A**). Putative ORF in circKEAP1 and the sequences of the putative ORF are shown. (**B**). Putative IRES activity in circKEAP1 was tested by dual luciferase activity assay. (**C**). U2OS cells were transfected with control vector, circKEAP1 ATG-mutated vector, circKEAP1 wild-type vector and linear KEAP1-259aa vector. CircKEAP1 expression was detected by qRT-PCR. (**D**). Total proteins from circKEAP1 or control plasmid-transfected U2OS cells were separated via SDS-PAGE. The differential gel bands between 25 kDa and 35 kDa were cut and subjected to LC-MS/MS. The identified KEAP1-259aa amino acids are shown. (**E**). KEAP1 and KEAP1-259aa expression were detected in eight paired normal and case-matched OS samples. (**F**). MG63 and U2OS cells were transfected with control or two circKEAP1 shRNAs. KEAP1 and KEAP1-259aa levels were characterized by western blotting. (**G**). KEAP1 and KEAP1-259aa expression were detected in control-, circKEAP1-, circKEAP1 IRSE mut-, and circKEAP1 ATG mut-transfected HOS and U2OS cells by western blotting. (**H**). Flag-tagged KEAP1-259aa was transfected into HOS and U2OS cells. Immunofluorescence staining using anti-Flag was performed to show KEAP1-259aa cellular localization (scale bars, 20 μm). Error bars represent three independent experiments. *, **, *** indicates significant differences compared with the empty vector group at a *p* value < 0.05, < 0.01, < 0.001, respectively

Based on the prediction results of the online database circRNADb, we found the circKEAP1 sequence to contain a short 777-nt ORF with an ATG initiation codon and an IRES at nucleotides 391–512, indicating circKEAP1 may encode a conserved 259-aa peptide (termed KEAP1-259aa) (Fig. 3A). Dual-luciferase assay results showed that the luciferase activity of the wild-type circKEAP1 IRES reporter was increased compared with that of a mutated IRES reporter (Fig. 3B). Following transfection, the Flag-circKEAP1 vector and Flag-circKEAP1-ATG mutant vector both increased circKEAP1 expression, while the control vector or linear circKEAP1-259aa transfection did not (Fig. 3C). Then, we transfected a circKEAP1-IRES mutant plasmid or circKEAP1 overexpression plasmid into HEK293T cells. Western blotting and silver staining of SDS-PAGE gel results showed that there was a protein band at an approximate molecular weight of 25 ˜ 35 kDa, which was subjected to LC-MS and found to have the sequence HSALRGQGTR, consistent with the amino acid sequence of KEAP1-259aa (Fig. 3D). To detect this polypeptide product, we used an antibody that recognized the middle amino acids 39–65 of KEAP1, which can also recognize KEAP1-259aa (Fig. 3SA). Western blotting was conducted to detect the levels of KEAP1-259aa and KEAP1 in 8 paired OS tissues and cell lines. KEAP1-259aa levels were lower in OS tissues compared with those in normal tissues (Fig. 3E). In addition, circKEAP1 expression level showed a clear positive correlation with KEAP1-259aa, but no significant correlation with KEAP1 (Fig. 3SB, C). Among the four OS cell lines, MG63 had the highest expression of KEAP1-259aa (Fig. 3SD). We found that circKEAP1 shRNAs reduced the expression of KEAP1-259aa in MG63 and U2OS cells, which had lower levels of KEAP1-259aa expression. Conversely, transfection of both circKEAP1 and linear KEAP1-259aa plasmids produced the predicted KEAP1-259aa band in U2OS and HOS cells, whereas transfection of the circKEAP1-IRES mutant plasmid or circKEAP1-ATG mutant vector did not (Fig. 3F, G). Immunofluorescence confirmed that KEAP1-259aa was located in the cytoplasm of cells transfected with the Flag-KEAP1-259aa plasmid (Fig. 3H). Overall, circKEAP1 encodes a novel KEAP1-259aa protein, in OS cells, whose expression depends on the circKEAP1 IRES.

### KEAP1-259aa exerts tumor suppressor functions

**Fig. 4:**
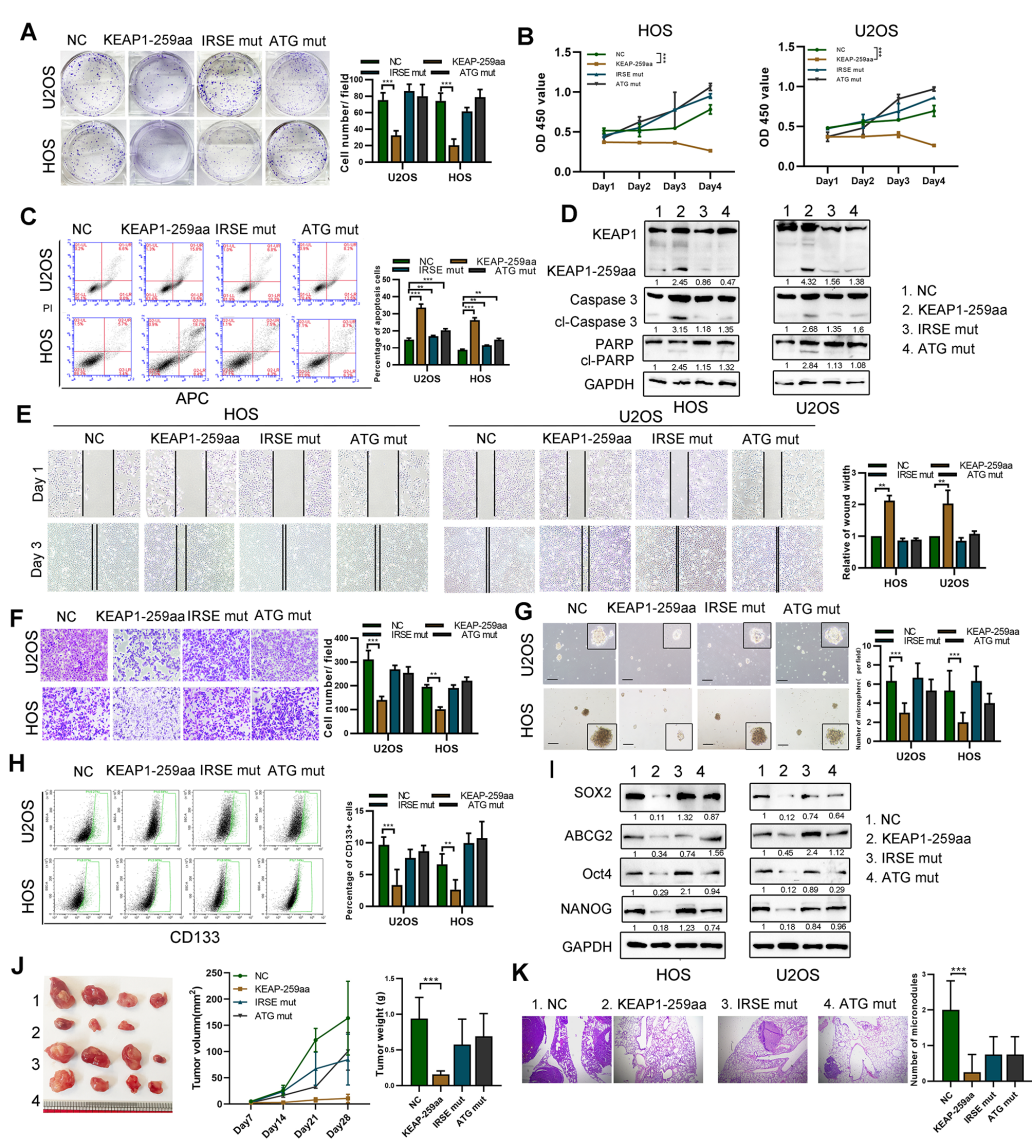
KEAP1-259aa exerts the biological function in OS cells. (**A**). OS cells were transfected with empty vector, circKEAP1 vector, ATG-mutated circKEAP1 vector and IRES-mutated circKEAP1 vector. Colony formation was performed in the indicated transfection groups. (**B**). Cell proliferation of the indicated cells was characterized by CCK-8 assay. (**C**). Apoptosis of the indicated cells was detected by flow cytometry. (**D**). Cell apoptosis of the indicated cells was characterized by western blotting assay. (**E**). Cell migration of the indicated cells was characterized by wound healing assay. (**F**). Cell migration of the indicated cells was characterized by transwell assay. (**G**). Tumor sphere assay was performed to detect stemness of the indicated cells (scale bar, 50 μm). (**H**). Flow cytometry assay was used to examine percentage of CD133 + cells in the indicated cells. (**I**). SOX2, ABCG2, Oct4 and Nanog expression in cells was characterized by western blotting. (**J**). The indicated cells (1 = NC, 2 = KEAP1-259aa, 3 = IRSE mut, 4 = ATG mut) were injected subcutaneously into nude mice (*n* = 4 per group) and tumor volume was measured every 7 days. After 28 days, tumors were collected and weighed. (**K**). The indicated cells were injected into nude mice (*n* = 4 per group) via tail vein and the lungs were harvested after 35 days. Representative image and H&E staining of mouse lung metastases is shown (scale bars, 200 μm). Error bars represent three independent experiments. *, **, *** indicates significant differences compared with the control group at a *p* value < 0.05, < 0.01, < 0.001, respectively

To explore the biological function of KEAP1-259aa, we established stable KEAP1-259aa-overexpression OS cells. Overexpressed of KEAP1-259aa inhibited OS cell proliferation and colony formation ability, and increased apoptosis (Fig. 4A-D). The KEAP1-259aa expression level negatively regulated cell migration, as detected by transwell and wound healing assay (Fig. 4E, F). Additionally, KEAP1-259aa expression inhibited OS cell stemness, based on non-adherent sphere formation, flow cytometry assay and western blotting (Fig. 4G-I). To further explore the possibility that circKEAP1 functions as a tumor suppressor in OS partly dependent on KEAP1-259aa, we restored KEAP1-259aa expression in stable circKEAP1 knockdown OS cells (Fig. 4SA). KEAP1-259aa overexpression reversed the malignant phenotypes induced by the stable knockdown of circKEAP1 (Fig. 4SB-F).

Then, we explored the role of KEAP1-259aa in vivo. Figure 4J shows that tumors generated from cells with stable overexpressing KEAP1-259aa had the smallest size and weight compared with cells transfected with control or the IRSE or ATG mutant plasmids. Similarly, we found that KEAP1-259aa-overexpression group had the lowest number of metastases in the lungs compared with the other groups (Fig. 4K). Collectively, these data show that KEAP1-259aa encoded by circKEAP1 inhibits cell proliferation, invasion and stemness of OS cells.

### KEAP1-259aa promotes vimentin proteasome degradation by binding with E3 ligase ARIH1

**Fig. 5:**
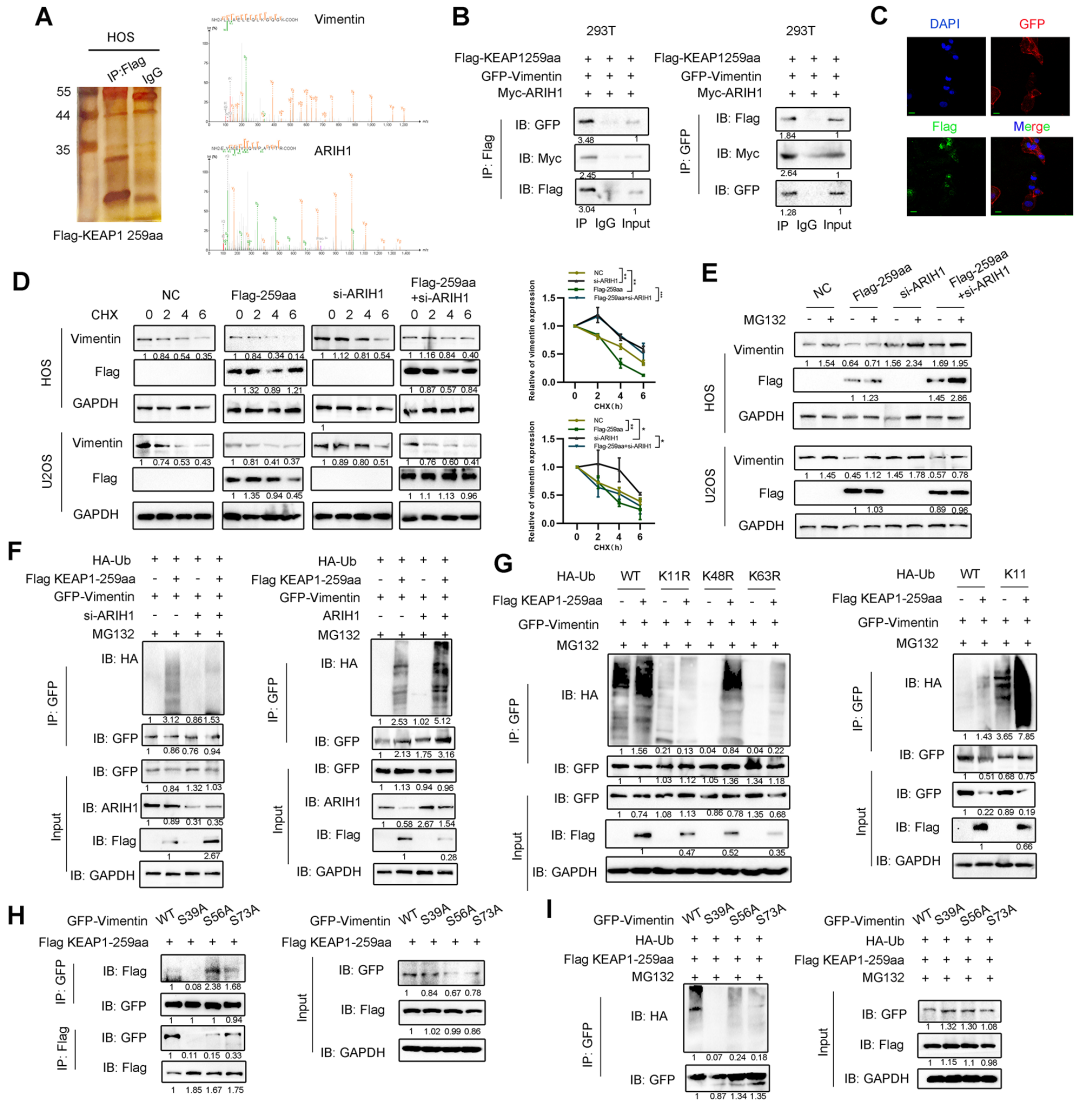
KEAP1-259aa interacts with ARIH1 to promote vimentin degradation. (**A**). Total immunoprecipitated proteins from Flag-KEAP1-259aa cells were separated via SDS-PAGE. Vimentin and ARIH1 were identified by LC/LC-MS. (**B**). Interaction of GFP-vimentin, Myc-ARIH1 and Flag-KEAP1-259aa were determined by co-IP assay. (**C**). Flag-tagged KEAP1-259aa and GFP-vimentin were co-transfected into MG63 cells and immunofluorescence staining was performed using anti-Flag and anti-GFP antibodies (scale bar, 20 μm). (**D**). Expression of vimentin in OS cells transfected with the indicated plasmid or siRNAs following the treatment with CHX (50 µg/ml). (**E**). Expression of vimentin was detected in OS cells transfected with the indicated plasmid or siRNAs following the treatment with MG132 (10 µM). (**F**). Ubiquitin of vimentin, in MG132 (10 µM)-treated cells, was detected by IP using GFP antibodies in the indicated cells followed by immunoblotting using HA antibodies. (**G**). Wild-type HA-Ub, K11R-, K48R- or K63R-mutant HA-Ub plasmids were transfected into OS cells. Wild-type HA-Ub, K11 HA-Ub plasmids were transfected into OS cells. Ubiquitin of vimentin was detected by IP assay in the treatment of MG132 (10 µM). (**H**). Wild-type GFP-vimentin, or vimentin S39A, S56A or S73A mutant plasmids were transfected into OS cells. The binding of vimentin and KEAP1-259aa was detected by co-IP assay. (**I**). Wild-type GFP-Vimentin, or vimentin S39A, S56A or S73A mutant plasmids were transfected into OS cells. Ubiquitin of vimentin was detected by IP assay in the treatment of MG132 (10 µM). Error bars represent three independent experiments. *, **, *** indicates significant differences compared with the control group at a *p* value < 0.05, < 0.01, < 0.001, respectively

To investigate the molecular mechanism by which KEAP1-259aa inhibits OS malignancy, we performed a mass spectrometry-based formaldehyde crosslinking assay in KEAP1-259aa OS cells. Gene Ontology (GO) analysis stated that KEAP1-259aa binding protein was associated with posttranslational modification (Figure S5A). The most abundantly recognized protein interacting with KEAP1-259aa in the ranking list are shown in Figure S5B and include vimentin and ARIH1, an ubiquitin transfer E3 ligase (Fig. 5A). Furthermore, co-immunoprecipitation assays showed that circKEAP1 could mutually interact with vimentin detected, supporting the direct interaction between exogenous KEAP1-259aa and vimentin as well as ARIH1 (Fig. 5B). Co-localization between KEAP1-259aa and vimentin as well as ARIH1 was confirmed by immunofluorescence staining (Fig. 5C and S5C).

Interestingly, KEAP-259aa overexpression decreased vimentin protein, but not mRNA expression (Fig. 5SD). To determine whether KEAP-259aa attenuated vimentin protein stability, we measured the half-life of vimentin and found a faster rate of vimentin degradation in KEAP-259aa-ovexpressing OS cells compared to control cells, whereas ARIH1 knockdown restore the expression of vimentin after treatment with cycloheximide (CHX), a protein synthesis inhibitor (Fig. 5D). Moreover, KEAP-259aa-mediated decrease in vimentin stability was reversed by MG132, a proteasome inhibitor (Fig. 5E). Immunoprecipitation experiments showed KEAP-259aa induced the polyubiquitination of vimentin (Fig. 5F). To detect the polyubiquitin chains linked toKEAP-259aa-polyubiquitinated vimentin, HEK293T cells were transiently transfected with GFP-vimentin together with HA-ubiquitin (wild-type or mutant) under conditions of Flag-KEAP-259aa overexpression or control followed by treatment with MG132. KEAP-259aa specifically promoted K11 polyubiquitination of vimentin and not any other chain types (Fig. 5G), indicating that KEAP-259aa mediated vimentin ubiquitination is dependent on K11 ubiquitin-linked chain formation.

To test whether the KEAP-259aa-mediated vimentin ubiquitin degradation is linked to phosphorylation of vimentin, we transfected plasmids encoding wild-type vimentin or individual phosphorylation site mutants into HEK293T cells. Interestingly, KEAP-259aa did not bind to the vimentin (S39A) mutant compared to wild-type protein and other serine mutants (Fig. 5H). Importantly, KEAP-259aa-induced ubiquitination of vimentin (S39A) was reduced compared with wild-type protein and other serine mutants (Fig. 5I). In sum, these results suggest that phosphorylation of vimentin at ser39 site is critical to promote KEAP-259aa-mediated ubiquitination and stability of vimentin.

### CircKEAP1 plays a tumor suppressor role through vimentin

**Fig. 6:**
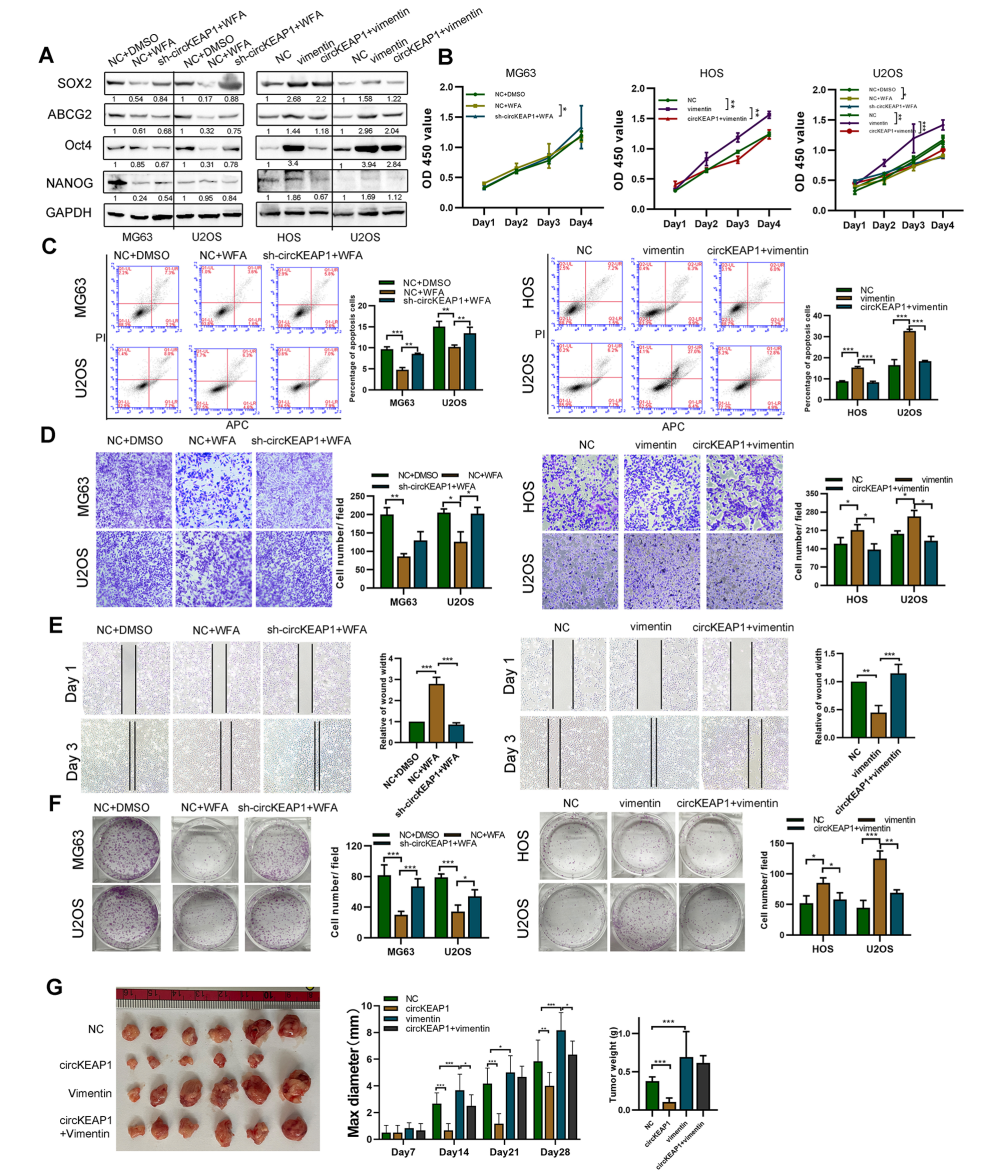
CircKEAP1 suppresses OS malignancy through decreasing vimentin levels. (**A**). OS cells were transfected with circKEAP1 shRNA and control vectors followed by treatment with WFA (5 µM). Control vectors, vimentin or both circKEAP1 and vimentin were transfected into OS cells. Caspase-3 and PARP expression were determined by western blotting. (**B**). Proliferation of the indicated OS cells was measured by CCK8 assay. (**C**). Apoptosis of the indicated OS cells was detected by flow cytometry. (**D**). Migration of the indicated OS cells was quantified by transwell assay. (**E**). Migration of the indicated OS cells was quantified by wound healing assay. (**F**). Colony formation ability of OS cells was determined. (**G**). The indicated cells were injected subcutaneously into nude mice (*n* = 6 per group) and tumor volume was measured every 7 days. After 28 days, tumors were collected and weighed. Error bars represent three independent experiments. *, **, *** indicates significant differences compared with the control group at a *p* value < 0.05, < 0.01, < 0.001, respectively

Vimentin has been proposed as a potential anti-tumor target for cancer therapy [20, 21]. Therefore, we treated OS cells with withaferin A (WFA), a vimentin inhibitor, and found that it reduced vimentin expression in the absence of changes in expression level of circKEAP1 (Fig. 6SA). As expected, the proliferation, colony formation, migration and stemness of circKEAP1 knockdown cells were decreased, while apoptosis was enhanced in cell lines treated with WFA. Furthermore, circKEAP1 did not exert tumor suppressor effects when cells were transfected with vimentin in vivo and in vitro (Fig. 6A-G and S6B-D). Taken together, these findings indicate that KEAP1-259aa encoded by circKEAP1 acted as a tumor suppressor by suppressing vimentin.

### CircKEAP1 is modulated by m6A RNA methylation

**Fig. 7:**
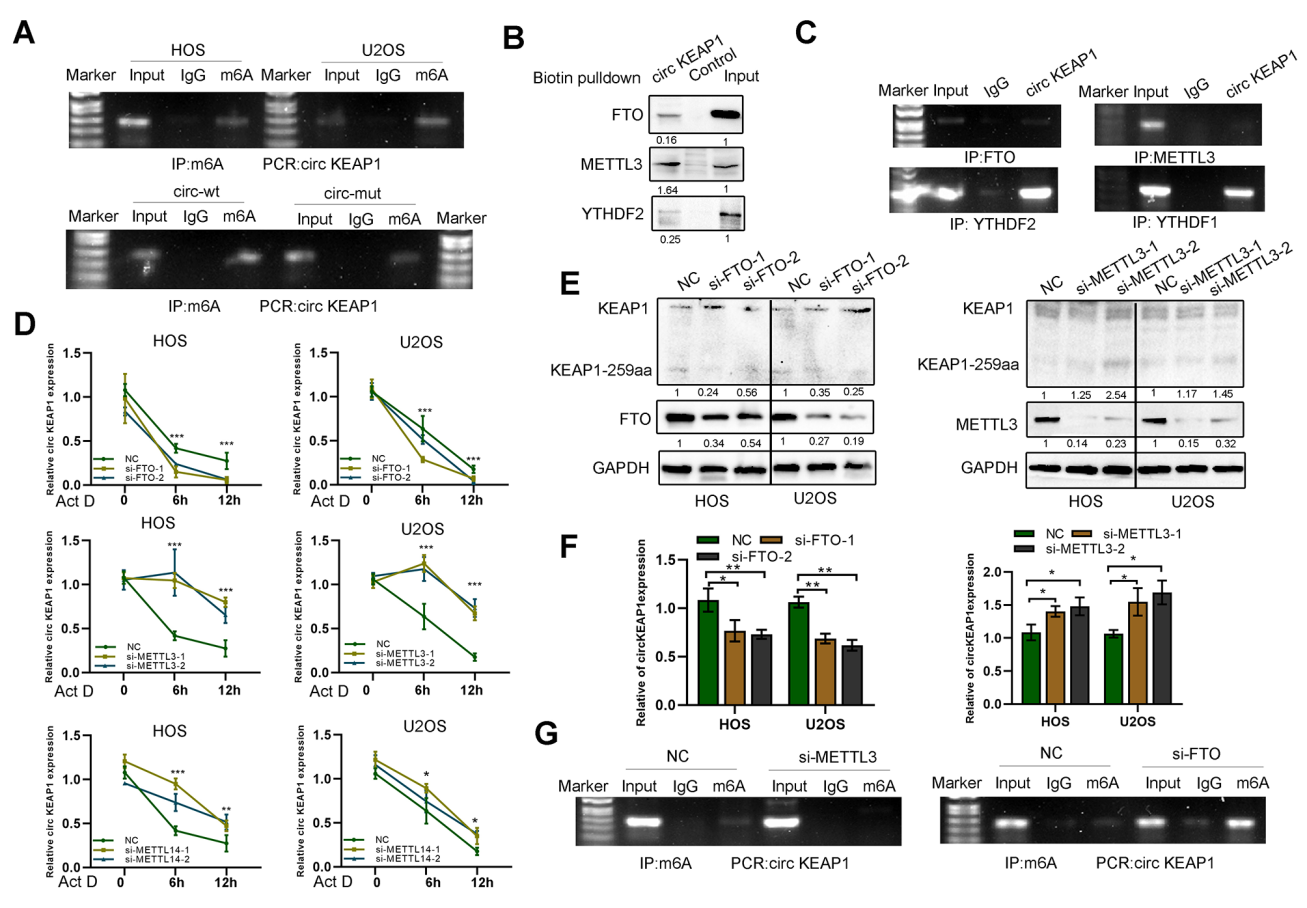
CircKEAP1 expression is modulated by m6A methylation. (**A**). MeRIP assays for m6A-modified circKEAP1 in HOS and U2OS cell lines, as well as cells over-expressing circKEAP1 with or without the m6A site mutated. (**B**). RNA pulldown assay was performed using a circKEAP1 or empty control probe and YTHDF2, METTL3, and FTO were detected by western blotting. (**C**). RIP assay and PCR analysis for circKEAP1 in U2OS cells using FTO, METTL3, YTHDF1/2 antibodies. (**D**). Expression of circKEAP1 in HOS and U2OS cells transfected with the indicated siRNAs under actinomycin D (5 µg/ml) treatment for 0, 6 and 12 h by qPCR. (**E**). Expression of KEAP1 was detected by western blotting in HOS and U2OS cells transfected with the indicated siRNAs. (**F**). QPCR analysis of circKEAP1 in HOS and U2OS cells transfected with control, si-METTL3 or si-FTO. (**G**). MeRIP assays for m6A-modified circKEAP1 in U2OS cells transfected with control, si-METTL3 or si-FTO. Error bars represent three independent experiments. *, **, *** indicates significant differences compared with 0 h or control group at a *p* value < 0.05, < 0.01, < 0.001, respectively

M6A, one of the most abundant modifications of RNA, plays a role in circRNA stability. To explore the detailed mechanisms controlling circKEAP1 levels in OS, we predicted m6A sites and found one m6A site in circKEAP1 predicted by SRAMP (http://www.cuilab.cn/sramp) (Figure S7A). Moreover, mutation of this m6A site (A565G) in circKEAP1 lowered circKEAP1 m6A level and increased circKEAP1stability (Fig. 7A and S7B). RNA pulldown and RIP assays indicated that circKEAP1 interacted with m6A modifiers, such as METTL3, FTO and YTHDF1/2 (Fig. 7B, C). Intriguingly, either METTL3 or METTL14 reduced the stability of circKEAP1 in HOS and U2OS cells, whereas circKEAP1 in FTO-overexpressing cells was more stable than NC-transfected cells under actinomycin D (Act D) treatment (Fig. 7D). Also, circKEAP1 and KEAP1-259aa were highly expressed in cells after transfection with si-METTL3 compared with the control group. CircKEAP1 and KEAP1-259aa expression was decreased after si-FTO transfection (Fig. 7E, F). Importantly, MeRIP assays showed m6A levels were reduced when adenosine methyltransferase METTL3 was silenced and were increased when FTO was silenced (Fig. 7G). Furthermore, we explored the expression of m6A modifiers in OS tissues and found the expressions of METTL3, METTL14, and YTHDF2 in OS were increased compared with normal tissues (Figure S7C). These results indicate that an m6A modification site at A565 exists on circKEAP1 and regulates circKEAP1 expression by decreasing its stability when methylated.

### CircKEAP1 triggers an immune defense response via the RIG-I pathway in OS cells

**Fig. 8:**
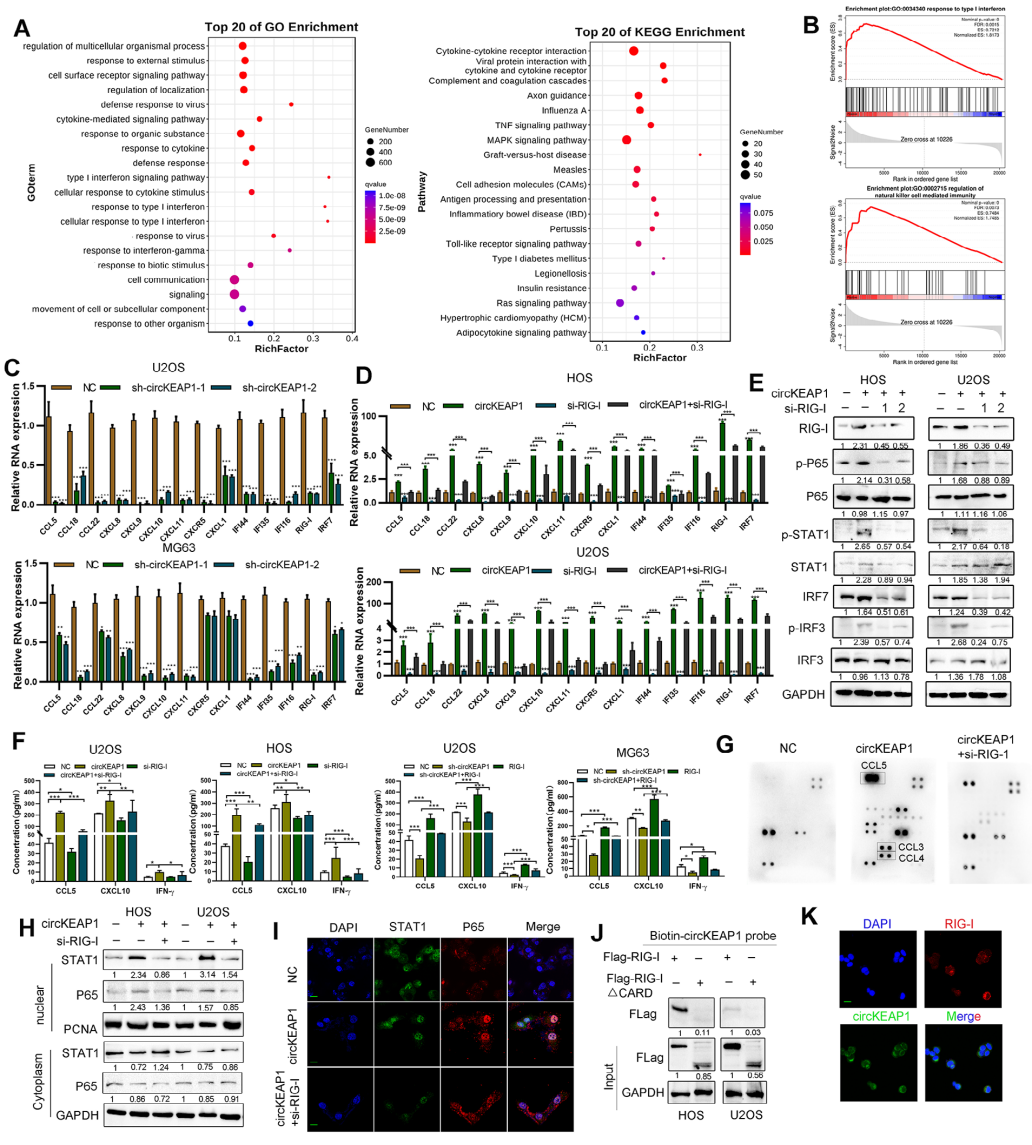
CircKEAP1 provokes cellular immune responses via RIG-I. (**A**). RNA sequencing was conducted in control and circKEAP1 overexpression cells. Kyoto Encyclopedia of Genes and Genomes (KEGG) and Gene Ontology (GO) pathway analyses were performed to find the most differentially-changed signaling pathways. (**B**). GSEA analysis for circKEAP1 compared to the control group. (**C**). Fold change of the indicated mRNAs in circKEAP1 knockdown and control OS cells was measured by qRT-PCR. (**D**). Expression levels of the indicated mRNAs in cells following RIG-I knockdown and/or circKEAP1-overexpression in U2OS and HOS cell lines. (**E**). Protein levels in HOS and U2OS cells with circKEAP1 overexpression and/or RIG-I knockdown were detected by western blotting. (**F**). Levels of CXCL10, CCL5 and IFNγ in the supernatants of cells was measured by ELISA following RIG-I knockdown or/and circKEAP1 overexpression in U2OS and HOS cell lines. (**G**). Expression of inflammatory cytokines in the supernatants of cells was characterized using a human inflammation antibody array. (**H**). Protein expression of STAT1 and P65 was detected by western blotting in the nuclear and cytoplasmic fractions of the indicated cells. (**I**). Cellular localization of STAT1 and P65 was detected by immunofluorescence staining (scale bars, 20 μm). (**J**). Biotin pulldown assay was performed using circKEAP1 probe and the anti-Flag was detected in U2OS and HOS cells. (**K**). FISH-IF staining was performed to show the cellular localization of circKEAP1 and RIG-I (scale bars, 20 μm). Error bars represent three independent experiments. *, **, *** indicates significant differences compared with the control or indicated group at a *p* value < 0.05, < 0.01, < 0.001, respectively

To investigate circKEAP1 downstream signaling pathways in OS, RNA-seq was performed. Among 1621 differentially-expressed genes, 1443 genes were upregulated and 178 genes were downregulated (Figure S8A). GO and KEGG pathway enrichment analysis showed that circKEAP1 was involved in type I interferon (IFN) and immunity signaling (Fig. 8A). Consistently, gene set enrichment analysis (GSEA) confirmed these findings (Fig. 8B). Based on qRT-PCR, expression of a panel of immune genes were decreased in OS cells following circKEAP1 knockdown, indicating that circKEAP1 elicits immune responses in OS cells (Fig. 8C). Interestingly, we detected a strong tendency towards statistical significance (*p* = 0.051) regarding the correlation with circKEAP1 expression and CD8 + T-cell density (Figure S8B). Previous research demonstrated that loss of RIG-I is involved in cancer immune escape [13], thus we speculated that circKEAP1 potently stimulated immune signaling mediated by RIG-I. RIG-I knockdown abrogated immune responses induced by circKEAP1 overexpression (Fig. 8D and S8C). We also noticed RIG-I was upregulated by circKEAP1. As shown in Fig. 8E, the protein expression of RIG-I, p-IRF3, p-IRF7, p-STAT1 and p-P65 were increased by circKEAP1, while total P65 and IRF3 expression were not affected by circKEAP1. ELISA and human inflammation antibody array results showed that circKEAP1 increased the levels of CXCL10, CCL5, and IFNγ in cell culture supernatant, while RIG-1 knockdown reduced the expression levels of these cytokines (Fig. 8F, G). Data also showed that circKEAP1 promoted while RIG-I knockdown abolished translocation of IRF3 and P65 from the cytoplasm into the nucleus, as detected by immunofluorescence and western blotting (Fig. 8H, I). To explore the potential mechanisms by which circKEAP1 regulate RIG-I, we performed RIP assay and FISH immunofluorescence experiments. Results showed that circKEAP1 bound to RIG-I via its CARD domain (Fig. 8J), and they were co-localized in the cytoplasm (Fig. 8K). These results indicate that overexpression of circKEAP1 leads to upregulation of immune response genes via RIG-1.

## Discussion

**Fig. 9:**
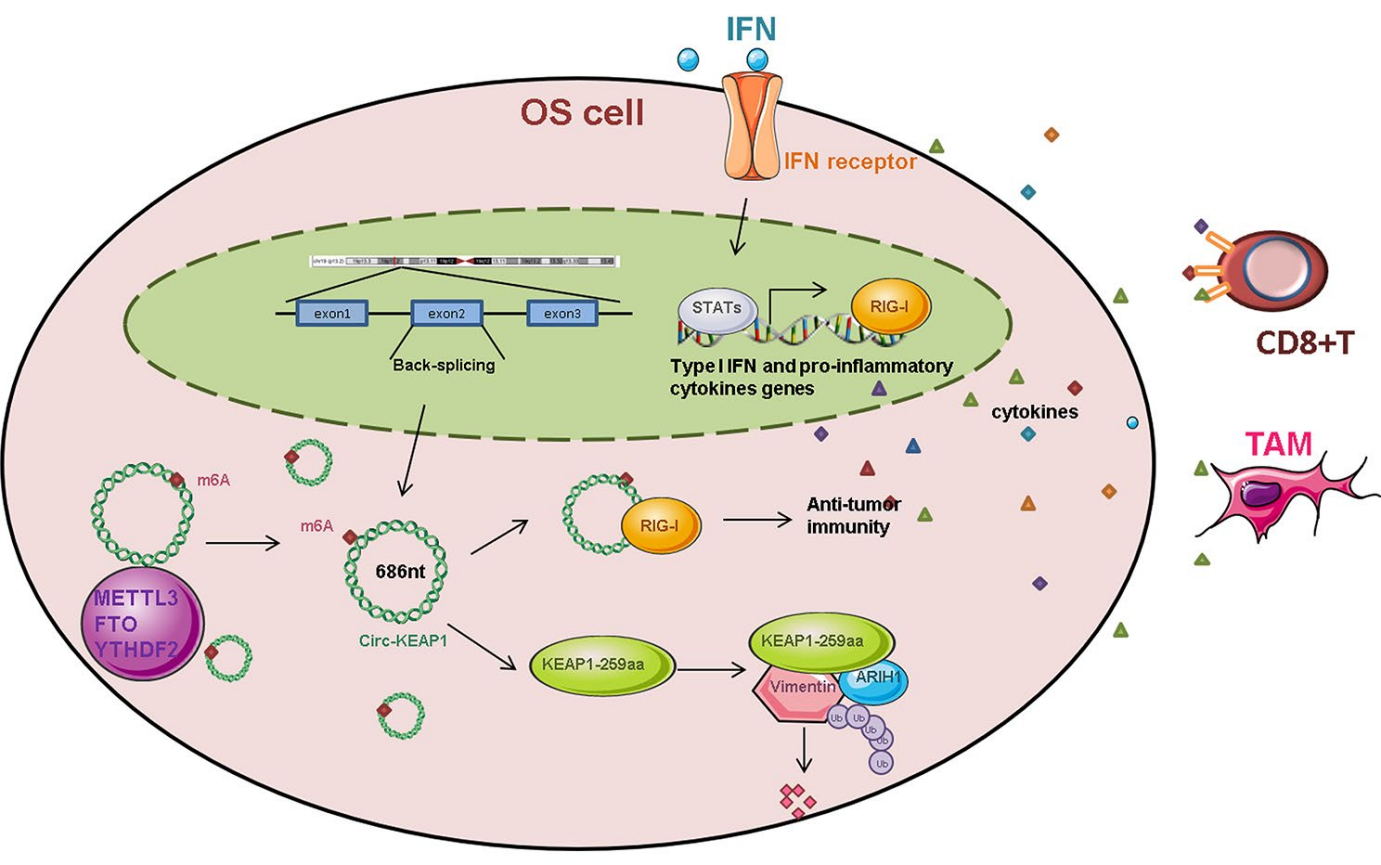
Schematic diagram of the project. In OS, circKEAP1 inhibits cell proliferation, migration and stemness by encoding a novel protein KEAP1-259aa, which mediated ubiquitination and stability of vimentin as well as triggers an immune defense response via the RIG-I pathway

We demonstrate a circRNA, circKEAP1, acts as a tumor suppressor in OS. CircKEAP1 is expressed at low levels in OS and is associated with OS prognosis. Functionally, circKEAP1 inhibits cancer stemness, invasion and proliferation. Mechanistically, circKEAP1 encodes a protein KEAP1-259aa, that binds to vimentin and promotes vimentin degradation by interacting with the E3 ligase ARIH1. More importantly, circKEAP1 activates anti-tumor immunity via the RIG-I signaling pathway. These results identify circKEAP1 as an important therapeutic target in OS (Fig. 9).

By high-throughput RNA sequencing and circRNA microarray analysis, an increasing number of circRNAs have been identified in regulatory signaling pathways linked to tumorigenesis. For example, circZKSCAN1 has been involved in stemness modulation by regulating FMRP protein expression and the CCAR1-β-catenin-WNT pathway in HCC cells [22]. Knockdown of circFAT1 impairs cancer stemness and inhibits head and neck squamous cell carcinoma tumorigenesis by reversing the tumor immunosuppressive microenvironment [23]. We identified the circRNA hsa_circ_0049271, termed circKEAP1, and showed it is expressed at low levels in OS compared with normal tissues. The decreased circKEAP1 expression in OS tissues correlates with worse patient survival. By gain- and loss-of-function experiments, we showed that circKEAP1 inhibits OS proliferation, migration and stemness of OS in vivo and in vitro.

The mechanism of circRNAs is their function as miRNA sponges, transcription modulators, and protein-binding RNA. In OS, most studies focused on the ceRNA function of circRNAs. Recent evidence has confirmed protein-coding circRNAs play a key role in tumorigenesis in HCC, glioblastoma, and colon cancer [24]. For example, circβ-catenin generates a novel isoform named β-catenin-370aa, that interacts with GSK3β to phosphorylate β-catenin and thus trigger degradation of β-catenin [25]. Circ-EIF6, encoding the novel peptide EIF6-224aa, enhances proliferation and metastasis of triple-negative breast cancer cells by activating the MYH9/Wnt/β-catenin pathway [26]. However, the roles of protein-coding circRNAs in OS have never been explored. In this paper, we demonstrate circKEAP1 encodes a 259 amino acid-long protein that plays a tumor suppressor role in OS. Functional experiments subsequently confirmed that KEAP1-259aa mediates the inhibitory effect of circKEAP1 in OS.

To further explore the mechanism of KEAP-259aa, we performed co-immunoprecipitation and showed that KEAP-259aa interacts with vimentin. Vimentin is a type III intermediate filament protein, and is considered a canonical biomarker of epithelial-to-mesenchymal transition (EMT) [27]. Increased vimentin expression is observed in several types of cancer, such as breast cancer, melanoma and lung cancer [28]. Moreover, EMT can transform vimentin-positive non-CSCs into cancer stem cells [29]. These findings highlight its central role in the regulation of cancer metastasis and stemness. A number of studies suggest that polyubiquitination is s critical post-translational modification that mediates vimentin expression and stability [30].For example, PD-L1 can bind to vimentin to inhibit vimentin ubiquitination degradation, resulting in pulmonary fibrosis [31]. Ubiquilin2 and myotubularin-1 recognize vimentin for degradation in skeletal muscle cells to avoid proteotoxic aggregate formation [32].In breast cancer, FERM domain-containing protein 3 (FRMD3) interacts with ubiquitin protein ligase E3A (UBE3A) to induce vimentin proteasomal degradation [33]. Trim56 and Trim16 are considered as ubiquitin ligase for vimentin to suppress ovarian cancer and lung adenocarcinoma progression, respectively [34, 35]. Our study finds that the expression and stabilization of vimentin proteins can be mediated by E3 ligand ARIH1 and crucial for KEAP-259aa-inhibited OS malignancy.

M6A modification regulate RNA biological processes, including RNA localization, splicing, translation and degradation [36]. It is well known that m6A binding proteins, such as METTL3/14, YTHDF1/2 and FTO, modulate m6A modification [37]. Moreover, circRNAs modified by m6A can be recognized by YTHDF1/2 and degraded by the RNase P/MRP complex, resulting altered circRNA stability and expression [38]. For example, METTL3 elevates m6A levels of hsa_circ_0029589 and decreases its expression, which promotes macrophage pyroptosis in acute coronary syndrome [39]. In this study, we first revealed that m6A modification is enriched in circKEAP1 by MeRIP and then hypothesized that the downregulation of circKEAP1 associated with m6A modification. By a series of experiments, we identified m6A in circKEAP1 and showed that the m6A level decreased circKEAP1 expression and stability, as well as was involved in the pathogenesis of OS. Consistent with the above findings, our study showed that METTL3, METTL14, and YTHDF2 are highly expressed in OS tissues, which may contribute to the low expression of circKEAP1. These results extended the knowledge about the effects of m6A modification on circKEAP1 expression.

As an RLR family member, RIG-I increases phosphorylation and nuclear translocation of IRF-3, IRF-7, NF-κB and STATs, and induces the expression of IFN-γ-induced genes and pro-inflammatory factors [16]. In addition to the key role of RIG-I in innate immune responses against viruses, RIG-I is also implicated in cancer development [40]. However, the role of RIG-I in cancer is not without controversy. RIG-I expression is downregulated and correlates with prolonged survival in HCC, but high RIG-I expression predicts poor clinical outcome for endometrial cancer [41, 42]. Here, we show circKEAP1 triggers an immune defense response by binding to RIG-I in OS cells. After activation of the RIG-I pathway, STAT1 and NF-κB translocate into the nucleus to increase the transcription and expression of IFNs and chemokines. Based on these in vitro experiments, we report that circKEAP1 participates in the infiltration of immune cells into the TME and inhibits OS progression. Our findings broaden our understanding of the mechanism in by which circRNAs activate anti-tumor immunity. In conclusion, our study shows that circKEAP1 inhibits OS malignancy by encoding a novel protein KEAP1-259aa, which may provide a potential therapeutic target in the treatment of OS.

### Electronic supplementary material

Below is the link to the electronic supplementary material.

Supplemental Figure 1: (**A**). Gene ontology analysis of most downregulated and upregulated circRNAs is shown. (**B**). The top differentially-expressed circRNAs were characterized in normal (*n* = 3) and OS tissues (*n* = 3). Error bars represent three independent experiments. *, **, *** indicates significant differences compared with the control group at a *p* value < 0.05, < 0.01, < 0.001, respectively

Supplemental Figure 2: (**A**). Expression of circKEAP1 was detected by qRT-PCR after transfection of circKEAP1 or two circKEAP1 shRNAs. (**B**). Expression of KEAP1 was detected by qRT-PCR and western blot after NC or circKEAP1 transfection. (**C**). CCK8 assay was performed to measure the proliferation following transfection of control, circKEAP1 or two circKEAP1 shRNA in OS cells. (**D**). Western blot analysis of SOX2, ABCG2, and NANOG in OS cells following transfection of circKEAP1, two circKEAP1 shRNAs or control. (**E**). Expression of cancer stem cells markers was detected by qRT-PCR in cells transfected with control, circKEAP1 or two circKEAP1 shRNAs. (**F**). Immunofluorescence staining using SOX2 and CD133 antibodies to detect their expression in cells transfected with two circKEAP1 shRNAs (scale bars, 20 μm). (**G**). Flow cytometry assay was used to detect CD133 + OS cells transfected with circKEAP1, two circKEAP1 shRNAs or control. (**H**). Representative image of tumorsphere formation of OS cells transfected with circKEAP1 or circKEAP1 shRNA as indicated (scale bar, 100 μm). Error bars represent three independent experiments. *, **, *** indicates significant differences compared with the control group at a *p* value < 0.05, < 0.01, < 0.001, respectively

Supplemental Figure 3: (**A**). Schematic diagram of the KEAP1 antibody detective residues. The KEAP1 antibody used in the study showing recognized both KEAP1 and KEAP1-259aa. (**B**). Correlation between circKEAP1 and KEAP1 expression in OS tissues was shown. (**C**). Correlation between circKEAP1 and KEAP1-259aa expression in OS tissues was shown. (**D**). KEAP1 and KEAP1-259aa expression were detected in OS cell lines by western blotting

Supplemental Figure 4: (**A**). The control, linear KEAP1-259aa and circKEAP1 shRNA + KEAP1-259aa plasmids were co-transfected into OS cells. The expression of SOX2, ABC2G, OCT4 and NANOG were measured by immunoblotting. (**B**). Cell proliferation was performed by CCK-8 assay in the indicated transfected groups. (**C**). Apoptosis was evaluated by flow cytometry in the indicated transfected groups. (**D**). Colony formation ability of the cells mentioned above was measured by colony formation assay. (**E**). Migration of the indicated cells was measured by transwell assay. (**F**). Migration ability of the indicated cells was measured by a wound healing assay. (**G**). Percentage of CD133 + cells in the indicated cells were measured by flow cytometry. (**H**). Effect of plasmids mentioned above on cell stemness was examined by tumorsphere formation (scale bar: 50 μm). Error bars represent three independent experiments. *, **, *** indicates significant differences compared with the control indicated or group at a *p* value < 0.05, < 0.01, < 0.001, respectively

Supplemental Figure 5: (**A**). LC-MS/MS was conducted to detect potential interacting proteins of KEAP1-259aa. GO analysis of binding proteins was performed. (**B**). The most abundance proteins in the ranking list. (**C**). Expression of vimentin and ARIH1 was detected by immunofluorescence staining in OS cells (scale bars, 20 μm). (**D**). Protein and mRNA expression of vimentin was detected by western blotting and qRT-PCR after KEAP1-259aa or circKEAP1 transfection

Supplemental Figure 6: (**A**) Expression of circKEAP1 was detected by qRT-PCR in OS cells treated with WFA (5 µM). Expression of vimentin was detected by western blotting in OS cells treated with different doses of WFA. (**B**) OS cells were transfected with circKEAP1 shRNA and control vectors followed by treatment with WFA. OS cells were transfected with circKEAP1, vimentin and control vectors. Expression of PARP was detected. (**C**) Flow cytometry was performed to determine the number of CD133 + OS cells. (**D**) Tumorsphere formation of OS cells are shown (scale bar, 50 μm). Error bars represent three independent experiments. *, **, *** indicates significant differences compared with the control group or indicated at a *p* value < 0.05, < 0.01, < 0.001, respectively

Supplemental Figure 7: (**A**). M6A site in circKEAP1 was predicted by RMBase v2.0. (**B**). Expression of circKEAP1 in U2OS cells over-expressing wild-type circKEAP1 or the m6A site-mutated circKEAP1 following treatment with actinomycin D (5 µg/ml) treatment for 0, 6 and 12 h. (**C**). Expression of the indicated protein was determined in 7 pairs of normal and OS tissues. Error bars represent three independent experiments. *, **, *** indicates significant differences compared with the 0 h group at a *p* value < 0.05, < 0.01, < 0.001, respectively

Supplemental Figure 8: (**A**). Differentially-expressed genes in U2OS cells with circKEAP1 versus control were identified by RNA-seq analysis. (**B**). Expression of CD8 in OS sample (*n* = 10) and correlation with circKEAP1 expression (scale bar, 200 μm). (**C**). Expression levels of the indicated mRNAs in cells following RIG-I knockdown and/or circKEAP1-overexpression U2OS and MG63 cell lines. Error bars represent three independent experiments. *, **, *** indicates significant differences compared with the control or indicated group at a *p* value < 0.05, < 0.01, < 0.001, respectively

Supplemental Tables 1 and 2

## Data Availability

All data generated or analyzed in this study are included in this article, and are available from the corresponding author on request.

## Abbreviations

Act D: Actinomycin D
ARIH: Ariadne-1 homolog
CCK-8: Cell counting kit-8
CHX: Cycloheximide
ChIP: Chromatin immunoprecipitation
CI: Confidence interval
CIS: Carcinoma in situ
CSC: Cancer stem cell
CXCL10: C-X-C motif chemokine 10
DAPI: 4’,6-diamidino-2-phenylindole
DEG: Differentially-expressed gene
DMEM: Dulbecco’ s modified eagle’ s medium
EMT: Epithelial-to-mesenchymal transition
FBS: Fetal bovine serum
FTO: Fat mass and obesity-associated protein
GAPDH: Glyceraldehyde-3-phosphate dehydrogenase
GO: Gene Ontology
HCC: Hepatocellular carcinoma
HDAC: Histone acetylation
HR: Hazard ratio
IHC: Immunohistochemistry
IFI27: IFN-α inducible protein 27
IFN: Interferon
IRSE: IP: Immunoprecipitation
KEAP1: Kelch-like ECH associated protein 1
KO: Knockout
NC: Negative control
m6A: N6-methyladenosine
MDA5: Melanoma differentiation-associated protein 5
MG132: Carbobenzoxy-Leu-Leu-leucinal
METTL3: Methyltransferase-like 3
NES: Normalized enrichment score
NF-kB: Nuclear factor kappa B
OS: Osteosarcoma
PBS: Phosphate-buffered saline
qRT-PCR: Quantitative reverse transcription polymerase chain reaction
3’UTRs: 3’ untranslated regions
RIG-I: Retinoic acid-inducible gene-I
RPMI: Roswell Park Memorial Institute. STAT:signal transducer and activator of transcription
YTHDF1/2: YT521-B homology domain

## References

[1] Lin Z, Xie X, Lu S, Liu T (2021). Noncoding RNAs in osteosarcoma: implications for drug resistance. Cancer Lett.

[2] Yan GN, Lv YF, Guo QN (2016). Advances in osteosarcoma stem cell research and opportunities for novel therapeutic targets. Cancer Lett.

[3] Zhou WY, Cai ZR, Liu J, Wang DS, Ju HQ, Xu RH (2020). Circular RNA: metabolism, functions and interactions with proteins. Mol Cancer.

[4] Soghli N, Qujeq D, Yousefi T, Soghli N (2020). The regulatory functions of circular RNAs in osteosarcoma. Genomics.

[5] Li Z, Li X, Xu D, Chen X, Li S, Zhang L, Chan MTV, Wu WKK (2021). An update on the roles of circular RNAs in osteosarcoma. Cell Prolif.

[6] Wu Y, Xie Z, Chen J, Chen J, Ni W, Ma Y, Huang K, Wang G, Wang J, Ma J (2019). Circular RNA circTADA2A promotes osteosarcoma progression and metastasis by sponging miR-203a-3p and regulating CREB3 expression. Mol Cancer.

[7] Shen S, Yao T, Xu Y, Zhang D, Fan S, Ma J (2020). CircECE1 activates energy metabolism in osteosarcoma by stabilizing c-Myc. Mol Cancer.

[8] Chen J, Liu G, Wu Y, Ma J, Wu H, Xie Z, Chen S, Yang Y, Wang S, Shen P (2019). CircMYO10 promotes osteosarcoma progression by regulating miR-370-3p/RUVBL1 axis to enhance the transcriptional activity of beta-catenin/LEF1 complex via effects on chromatin remodeling. Mol Cancer.

[9] Lee Y, Choe J, Park OH, Kim YK (2020). Molecular mechanisms driving mRNA degradation by m(6)a modification. Trends Genet.

[10] Wang X, Ma R, Zhang X, Cui L, Ding Y, Shi W, Guo C, Shi Y (2021). Crosstalk between N6-methyladenosine modification and circular RNAs: current understanding and future directions. Mol Cancer.

[11] Zhang L, Hou C, Chen C, Guo Y, Yuan W, Yin D, Liu J, Sun Z (2020). The role of N(6)-methyladenosine (m(6)A) modification in the regulation of circRNAs. Mol Cancer.

[12] Zhang W, Wang L, Zhang P, Zhang Q (2021). m6A regulators are associated with osteosarcoma metastasis and have prognostic significance: a study based on public databases. Medicine.

[13] Xu S, Jin T, Weng J (2022). Endothelial cells as a key cell type for innate immunity: a focused review on RIG-I signaling pathway. Front Immunol.

[14] Vilgelm AE, Richmond A (2019). Chemokines modulate Immune Surveillance in Tumorigenesis, Metastasis, and response to Immunotherapy. Front Immunol.

[15] Iurescia S, Fioretti D, Rinaldi M. The Innate Immune Signalling pathways: turning RIG-I sensor activation against Cancer. Cancers 2020, 12(11). 10.3390/cancers12113158 PMC7693898 33121210

[16] Rameshbabu S, Labadie BW, Argulian A, Patnaik A. Targeting Innate Immunity in Cancer Therapy. Vaccines 2021, 9(2). 10.3390/vaccines9020138 PMC7916062 33572196

[17] Zhang Y, Liu Z, Yang X, Lu W, Chen Y, Lin Y, Wang J, Lin S, Yun JP (2021). H3K27 acetylation activated-COL6A1 promotes osteosarcoma lung metastasis by repressing STAT1 and activating pulmonary cancer-associated fibroblasts. Theranostics.

[18] Zimmerman SG, Peters NC, Altaras AE, Berg CA (2013). Optimized RNA ISH, RNA FISH and protein-RNA double labeling (IF/FISH) in Drosophila ovaries. Nat Protoc.

[19] Saygin C, Matei D, Majeti R, Reizes O, Lathia JD (2019). Targeting Cancer Stemness in the clinic: from hype to Hope. Cell Stem Cell.

[20] Peuhu E, Virtakoivu R, Mai A, Warri A, Ivaska J (2017). Epithelial vimentin plays a functional role in mammary gland development. Development.

[21] Bollong MJ, Pietila M, Pearson AD, Sarkar TR, Ahmad I, Soundararajan R, Lyssiotis CA, Mani SA, Schultz PG, Lairson LL (2017). A vimentin binding small molecule leads to mitotic disruption in mesenchymal cancers. Proc Natl Acad Sci USA.

[22] Zhu YJ, Zheng B, Luo GJ, Ma XK, Lu XY, Lin XM, Yang S, Zhao Q, Wu T, Li ZX (2019). Circular RNAs negatively regulate cancer stem cells by physically binding FMRP against CCAR1 complex in hepatocellular carcinoma. Theranostics.

[23] Jia L, Wang Y, Wang CY (2021). circFAT1 promotes Cancer Stemness and Immune Evasion by promoting STAT3 activation. Adv Sci.

[24] Lei M, Zheng G, Ning Q, Zheng J, Dong D (2020). Translation and functional roles of circular RNAs in human cancer. Mol Cancer.

[25] Liang WC, Wong CW, Liang PP, Shi M, Cao Y, Rao ST, Tsui SK, Waye MM, Zhang Q, Fu WM (2019). Translation of the circular RNA circbeta-catenin promotes liver cancer cell growth through activation of the wnt pathway. Genome Biol.

[26] Li Y, Wang Z, Su P, Liang Y, Li Z, Zhang H, Song X, Han D, Wang X, Liu Y (2022). circ-EIF6 encodes EIF6-224aa to promote TNBC progression via stabilizing MYH9 and activating the Wnt/beta-catenin pathway. Mol Therapy: J Am Soc Gene Therapy.

[27] Kuburich NA, den Hollander P, Pietz JT, Mani SA (2022). Vimentin and cytokeratin: good alone, bad together. Sem Cancer Biol.

[28] Liu CY, Lin HH, Tang MJ, Wang YK (2015). Vimentin contributes to epithelial-mesenchymal transition cancer cell mechanics by mediating cytoskeletal organization and focal adhesion maturation. Oncotarget.

[29] Guen VJ, Chavarria TE, Kroger C, Ye X, Weinberg RA, Lees JA (2017). EMT programs promote basal mammary stem cell and tumor-initiating cell stemness by inducing primary ciliogenesis and hedgehog signaling. Proc Natl Acad Sci USA.

[30] Snider NT, Omary MB (2014). Post-translational modifications of intermediate filament proteins: mechanisms and functions. Nat Rev Mol Cell Biol.

[31] Li Q, Deng MS, Wang RT, Luo H, Luo YY, Zhang DD, Chen KJ, Cao XF, Yang GM, Zhao TM (2023). PD-L1 upregulation promotes drug-induced pulmonary fibrosis by inhibiting vimentin degradation. Pharmacol Res.

[32] Gavriilidis C, Laredj L, Solinhac R, Messaddeq N, Viaud J, Laporte J, Sumara I, Hnia K (2018). The MTM1-UBQLN2-HSP complex mediates degradation of misfolded intermediate filaments in skeletal muscle. Nat Cell Biol.

[33] Shao W, Li J, Piao Q, Yao X, Li M, Wang S, Song Z, Sun Y, Zheng L, Wang G (2023). FRMD3 inhibits the growth and metastasis of breast cancer through the ubiquitination-mediated degradation of vimentin and subsequent impairment of focal adhesion. Cell Death Dis.

[34] Zhao L, Zhang P, Su XJ, Zhang B (2018). The ubiquitin ligase TRIM56 inhibits ovarian cancer progression by targeting vimentin. J Cell Physiol.

[35] Tian H, Lian R, Li Y, Liu C, Liang S, Li W, Tao T, Wu X, Ye Y, Yang X (2020). AKT-induced lncRNA VAL promotes EMT-independent metastasis through diminishing Trim16-dependent vimentin degradation. Nat Commun.

[36] Xu T, He B, Sun H, Xiong M, Nie J, Wang S, Pan Y (2022). Novel insights into the interaction between N6-methyladenosine modification and circular RNA. Mol Therapy Nucleic Acids.

[37] Lin H, Wang Y, Wang P, Long F, Wang T (2022). Mutual regulation between N6-methyladenosine (m6A) modification and circular RNAs in cancer: impacts on therapeutic resistance. Mol Cancer.

[38] Park OH, Ha H, Lee Y, Boo SH, Kwon DH, Song HK, Kim YK (2019). Endoribonucleolytic cleavage of m(6)A-Containing RNAs by RNase P/MRP complex. Mol Cell.

[39] Guo M, Yan R, Ji Q, Yao H, Sun M, Duan L, Xue Z, Jia Y (2020). IFN regulatory Factor-1 induced macrophage pyroptosis by modulating m6A modification of circ_0029589 in patients with acute coronary syndrome. Int Immunopharmacol.

[40] Onomoto K, Onoguchi K, Yoneyama M (2021). Regulation of RIG-I-like receptor-mediated signaling: interaction between host and viral factors. Cell Mol Immunol.

[41] Beyer S, Muller L, Mitter S, Keilmann L, Meister S, Buschmann C, Kraus F, Topalov NE, Czogalla B, Trillsch F (2023). High RIG-I and EFTUD2 expression predicts poor survival in endometrial cancer. J Cancer Res Clin Oncol.

[42] Hou J, Zhou Y, Zheng Y, Fan J, Zhou W, Ng IO, Sun H, Qin L, Qiu S, Lee JM (2014). Hepatic RIG-I predicts survival and interferon-alpha therapeutic response in hepatocellular carcinoma. Cancer Cell.

