# Supplementary figures and images for: A tumor suppressor protein encoded by circKEAP1 inhibits osteosarcoma cell stemness and metastasis by promoting vimentin proteasome degradation and activating anti-tumor immunity
Source: J Exp Clin Cancer Res. 2024 Feb 21;43:52. doi: 10.1186/s13046-024-02971-7 (PMC10880370; doi:10.1186/s13046-024-02971-7)

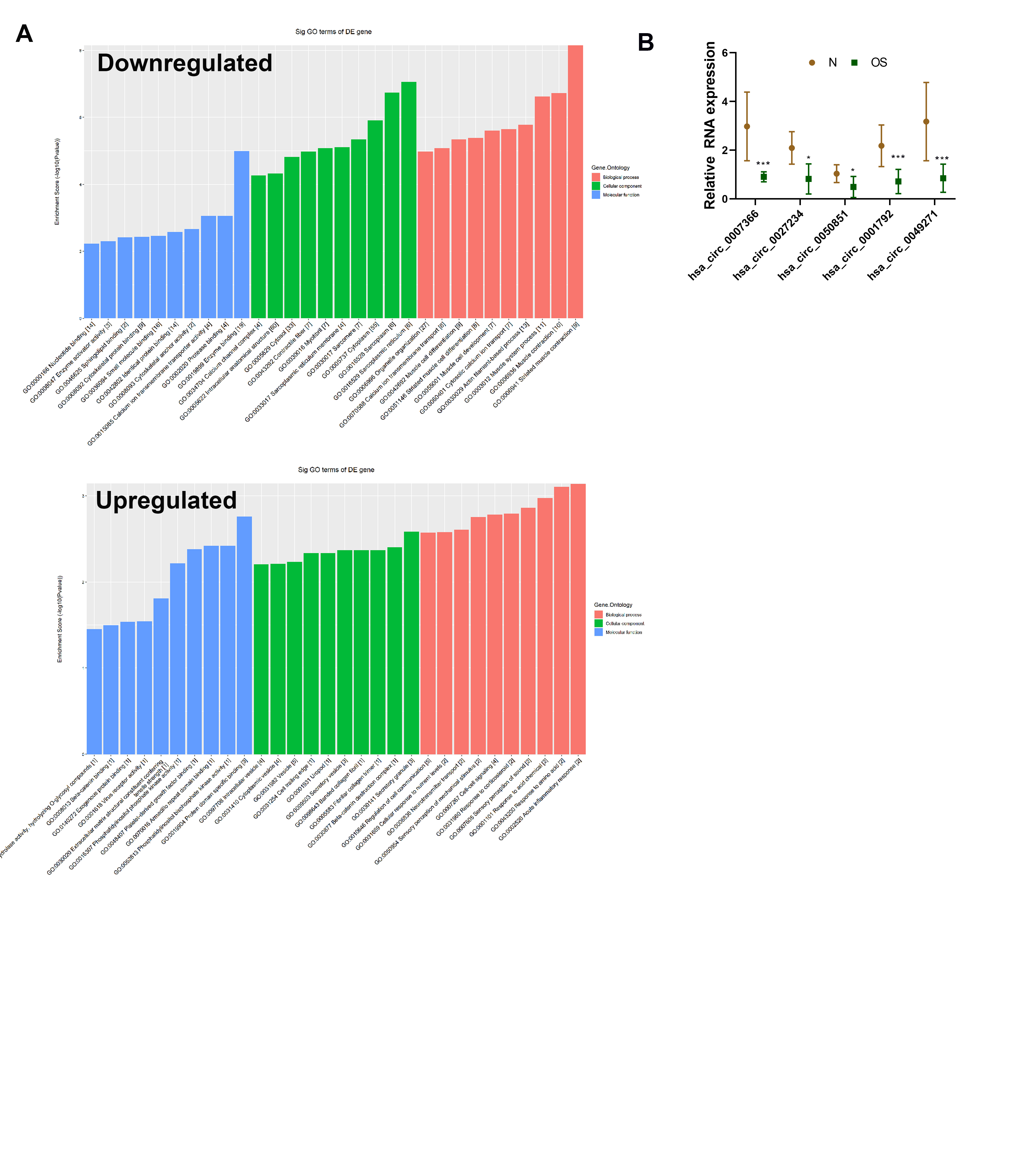

Supplement: Supplementary file 1 — Supplemental Figure 1: (A). Gene ontology analysis of most downregulated and upregulated circRNAs is shown. (B). The top differentially-expressed circRNAs were characterized in normal (n = 3) and OS tissues (n = 3). Error bars represent three independent experiments. *, **, *** indicates significant differences compared with the control group at a p value < 0.05, < 0.01, < 0.001, respectively [file 13046_2024_2971_MOESM1_ESM.jpg]

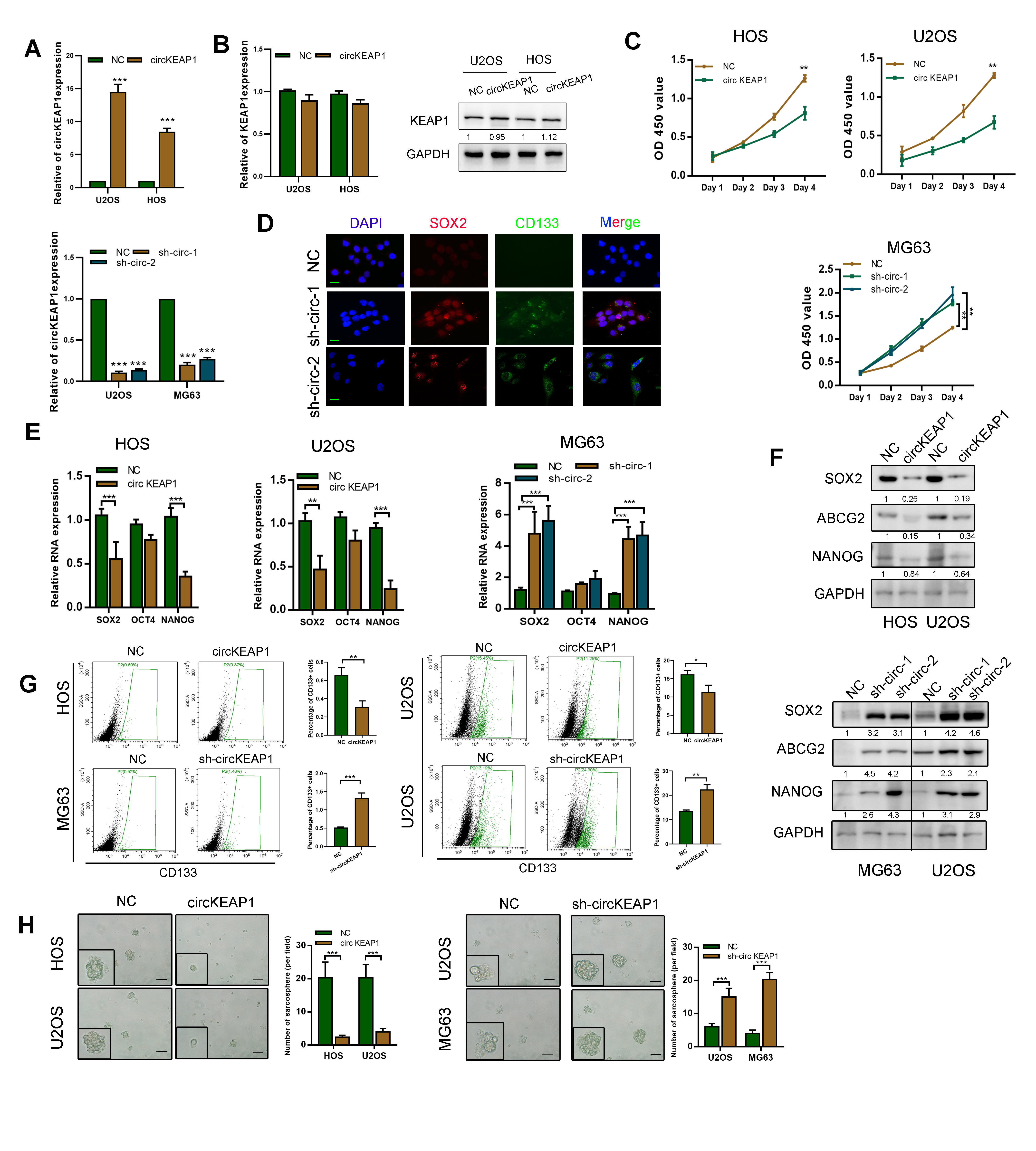

Supplement: Supplementary file 2 — Supplemental Figure 2: (A). Expression of circKEAP1 was detected by qRT-PCR after transfection of circKEAP1 or two circKEAP1 shRNAs. (B). Expression of KEAP1 was detected by qRT-PCR and western blot after NC or circKEAP1 transfection. (C). CCK8 assay was performed to measure the proliferation following transfection of control, circKEAP1 or two circKEAP1 shRNA in OS cells. (D). Western blot analysis of SOX2, ABCG2, and NANOG in OS cells following transfection of circKEAP1, two circKEAP1 shRNAs or control. (E). Expression of cancer stem cells markers was detected by qRT-PCR in cells transfected with control, circKEAP1 or two circKEAP1 shRNAs. (F). Immunofluorescence staining using SOX2 and CD133 antibodies to detect their expression in cells transfected with two circKEAP1 shRNAs (scale bars, 20 μm). (G). Flow cytometry assay was used to detect CD133 + OS cells transfected with circKEAP1, two circKEAP1 shRNAs or control. (H). Representative image of tumorsphere formation of OS cells transfected with circKEAP1 or circKEAP1 shRNA as indicated (scale bar, 100 μm). Error bars represent three independent experiments. *, **, *** indicates significant differences compared with the control group at a p value < 0.05, < 0.01, < 0.001, respectively [file 13046_2024_2971_MOESM2_ESM.jpg]

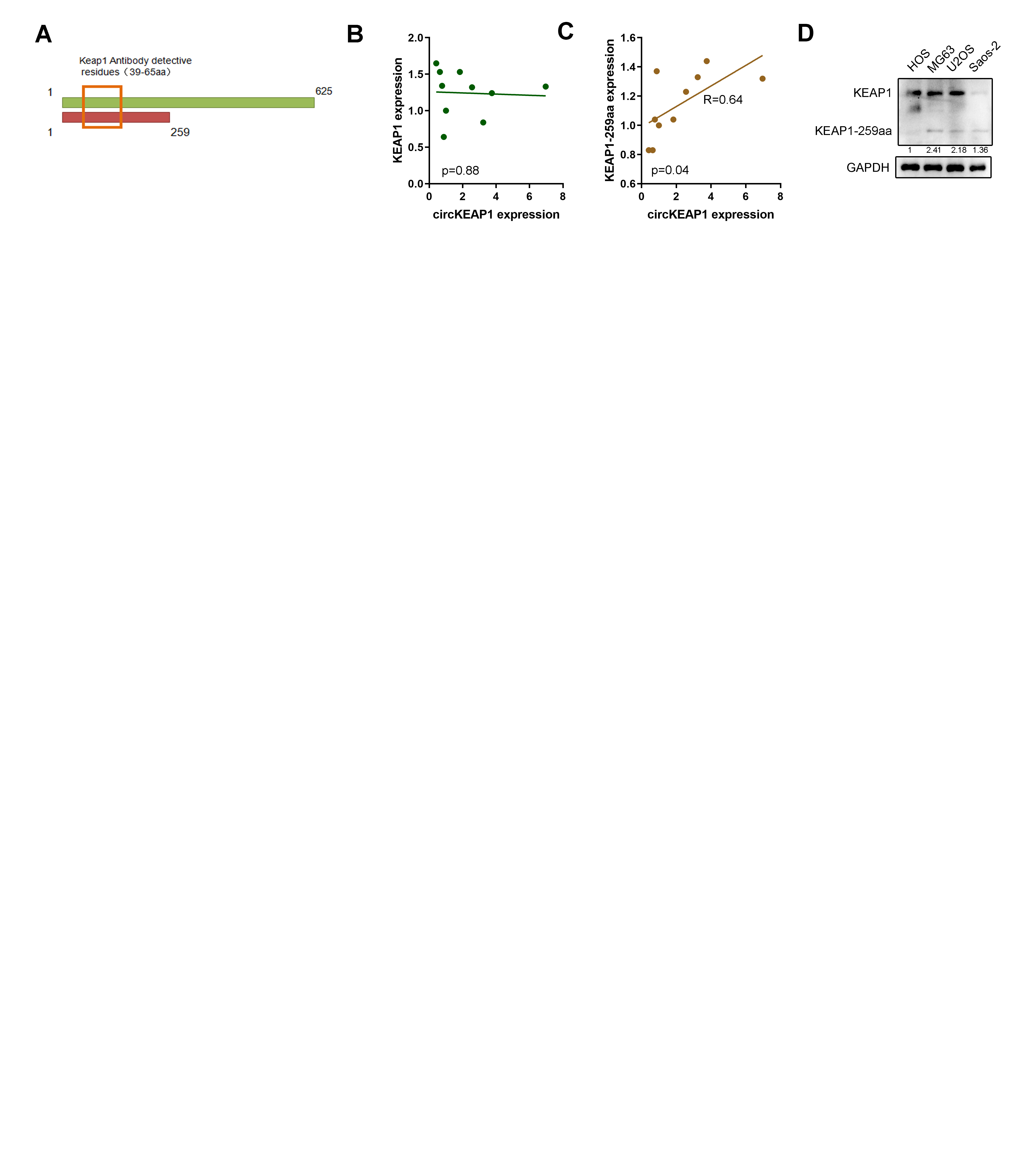

Supplement: Supplementary file 3 — Supplemental Figure 3: (A). Schematic diagram of the KEAP1 antibody detective residues. The KEAP1 antibody used in the study showing recognized both KEAP1 and KEAP1-259aa. (B). Correlation between circKEAP1 and KEAP1 expression in OS tissues was shown. (C). Correlation between circKEAP1 and KEAP1-259aa expression in OS tissues was shown. (D). KEAP1 and KEAP1-259aa expression were detected in OS cell lines by western blotting [file 13046_2024_2971_MOESM3_ESM.jpg]

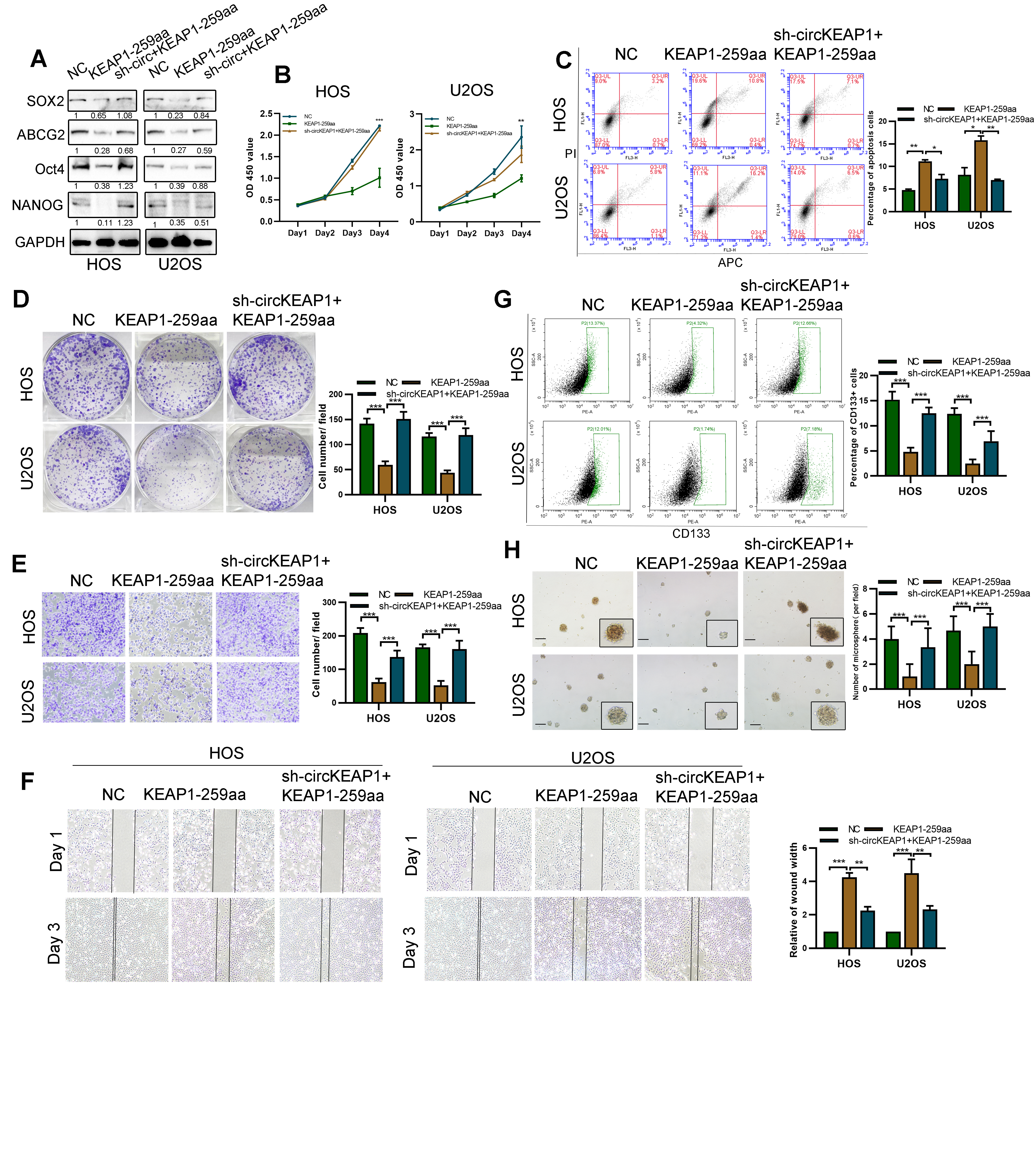

Supplement: Supplementary file 4 — Supplemental Figure 4: (A). The control, linear KEAP1-259aa and circKEAP1 shRNA + KEAP1-259aa plasmids were co-transfected into OS cells. The expression of SOX2, ABC2G, OCT4 and NANOG were measured by immunoblotting. (B). Cell proliferation was performed by CCK-8 assay in the indicated transfected groups. (C). Apoptosis was evaluated by flow cytometry in the indicated transfected groups. (D). Colony formation ability of the cells mentioned above was measured by colony formation assay. (E). Migration of the indicated cells was measured by transwell assay. (F). Migration ability of the indicated cells was measured by a wound healing assay. (G). Percentage of CD133 + cells in the indicated cells were measured by flow cytometry. (H). Effect of plasmids mentioned above on cell stemness was examined by tumorsphere formation (scale bar: 50 μm). Error bars represent three independent experiments. *, **, *** indicates significant differences compared with the control indicated or group at a p value < 0.05, < 0.01, < 0.001, respectively [file 13046_2024_2971_MOESM4_ESM.jpg]

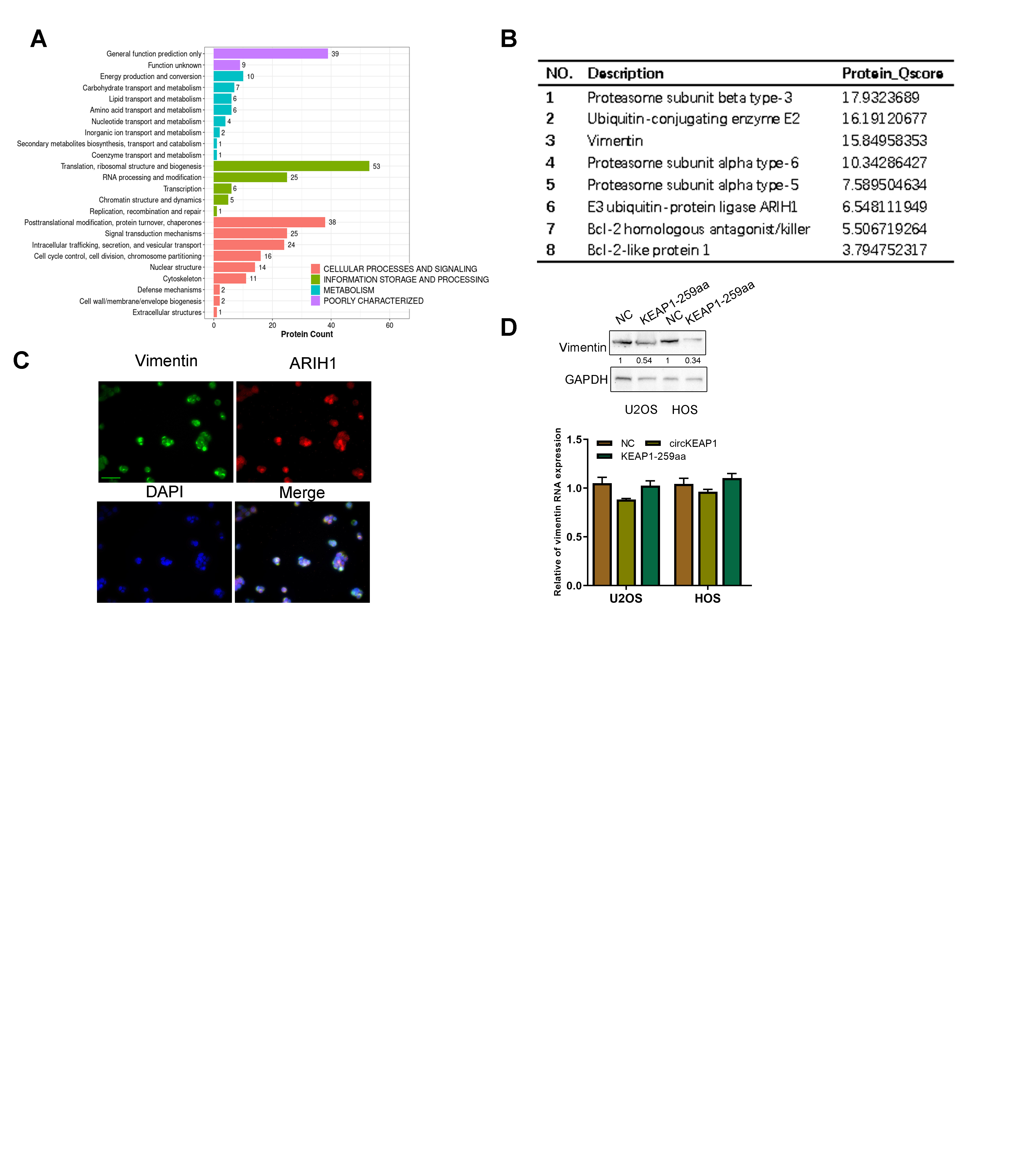

Supplement: Supplementary file 5 — Supplemental Figure 5: (A). LC-MS/MS was conducted to detect potential interacting proteins of KEAP1-259aa. GO analysis of binding proteins was performed. (B). The most abundance proteins in the ranking list. (C). Expression of vimentin and ARIH1 was detected by immunofluorescence staining in OS cells (scale bars, 20 μm). (D). Protein and mRNA expression of vimentin was detected by western blotting and qRT-PCR after KEAP1-259aa or circKEAP1 transfection [file 13046_2024_2971_MOESM5_ESM.jpg]

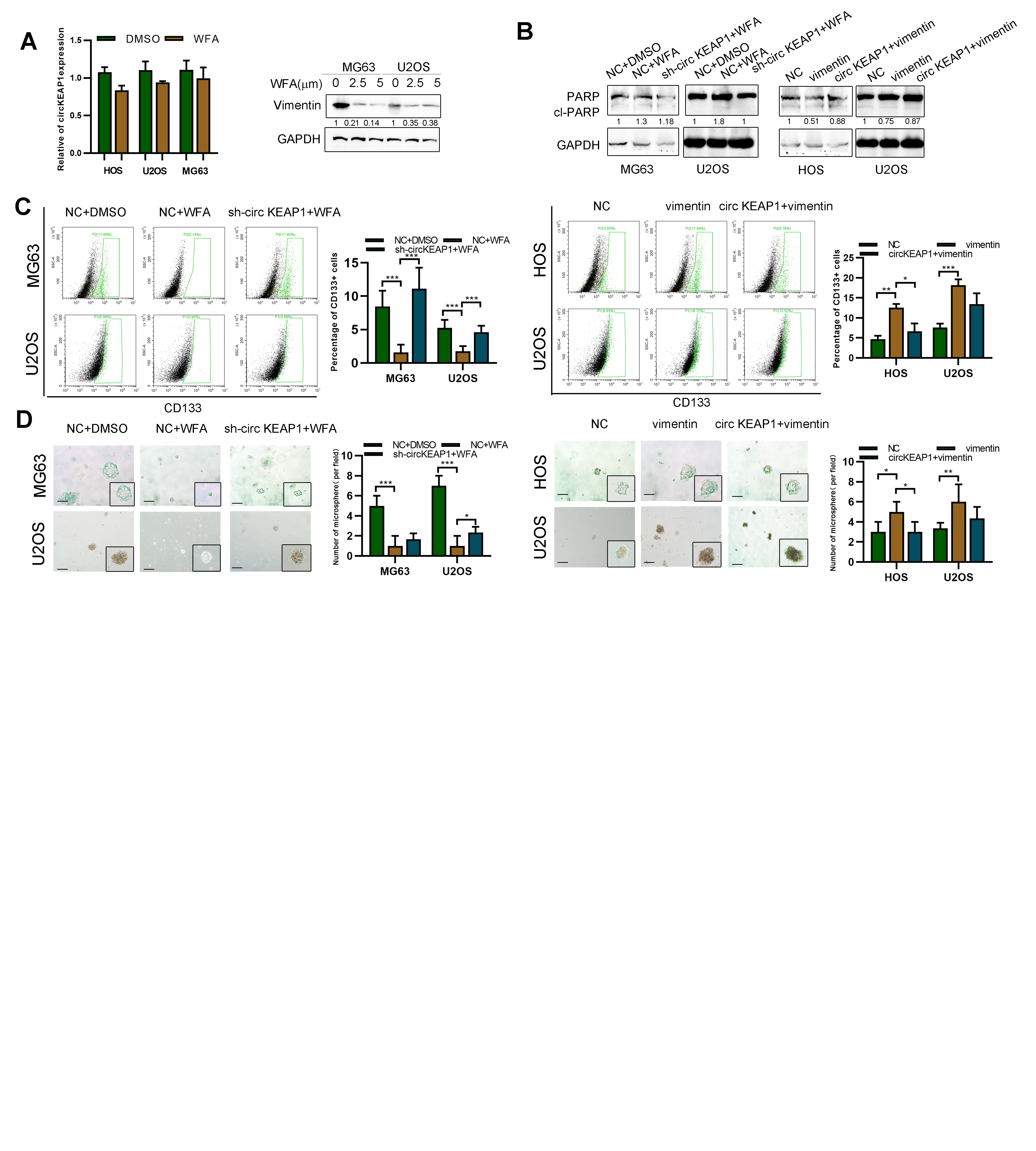

Supplement: Supplementary file 6 — Supplemental Figure 6: (A) Expression of circKEAP1 was detected by qRT-PCR in OS cells treated with WFA (5 µM). Expression of vimentin was detected by western blotting in OS cells treated with different doses of WFA. (B) OS cells were transfected with circKEAP1 shRNA and control vectors followed by treatment with WFA. OS cells were transfected with circKEAP1, vimentin and control vectors. Expression of PARP was detected. (C) Flow cytometry was performed to determine the number of CD133 + OS cells. (D) Tumorsphere formation of OS cells are shown (scale bar, 50 μm). Error bars represent three independent experiments. *, **, *** indicates significant differences compared with the control group or indicated at a p value < 0.05, < 0.01, < 0.001, respectively [file 13046_2024_2971_MOESM6_ESM.jpg]

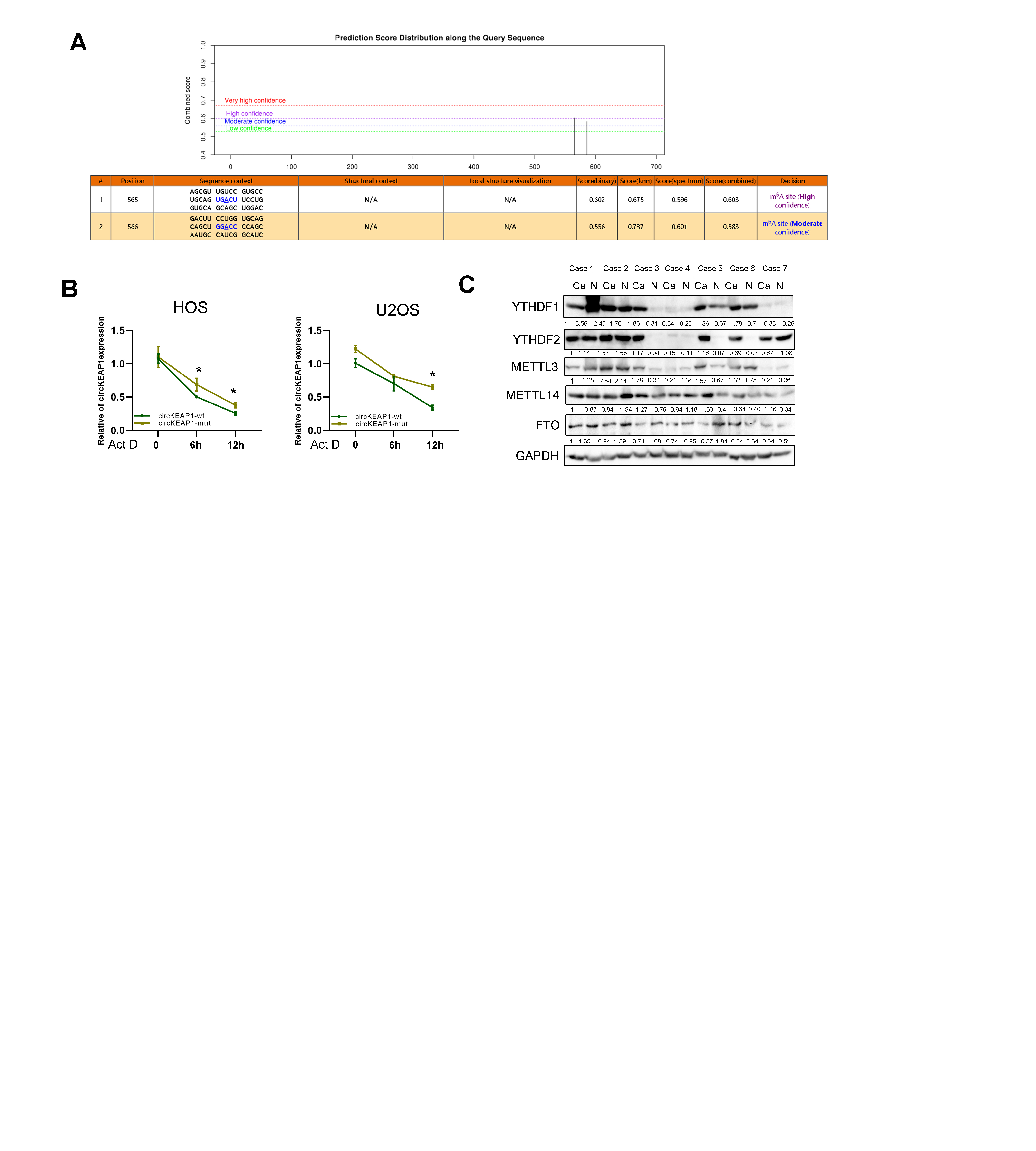

Supplement: Supplementary file 7 — Supplemental Figure 7: (A). M6A site in circKEAP1 was predicted by RMBase v2.0. (B). Expression of circKEAP1 in U2OS cells over-expressing wild-type circKEAP1 or the m6A site-mutated circKEAP1 following treatment with actinomycin D (5 µg/ml) treatment for 0, 6 and 12 h. (C). Expression of the indicated protein was determined in 7 pairs of normal and OS tissues. Error bars represent three independent experiments. *, **, *** indicates significant differences compared with the 0 h group at a p value < 0.05, < 0.01, < 0.001, respectively [file 13046_2024_2971_MOESM7_ESM.jpg]

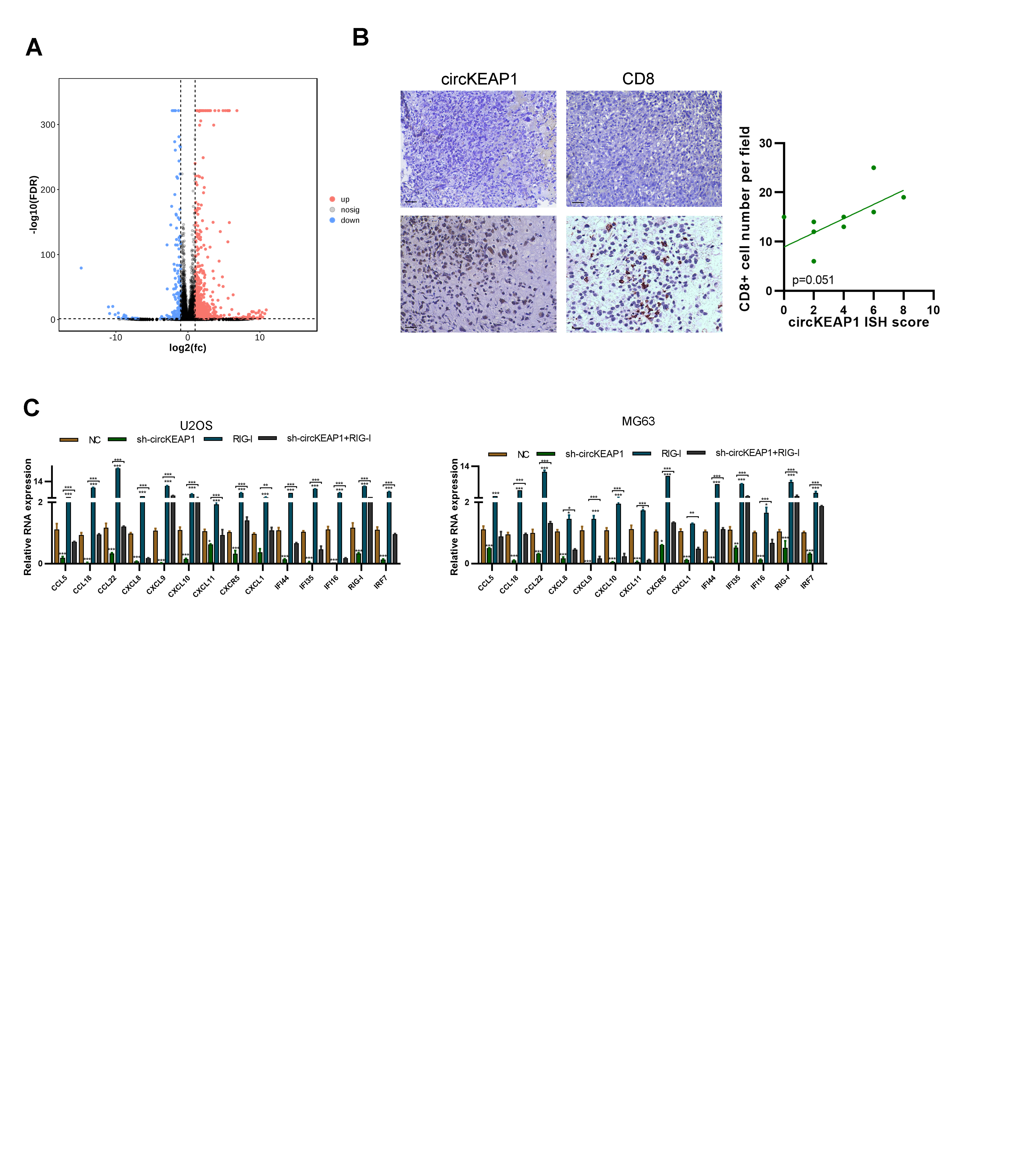

Supplement: Supplementary file 8 — Supplemental Figure 8: (A). Differentially-expressed genes in U2OS cells with circKEAP1 versus control were identified by RNA-seq analysis. (B). Expression of CD8 in OS sample (n = 10) and correlation with circKEAP1 expression (scale bar, 200 μm). (C). Expression levels of the indicated mRNAs in cells following RIG-I knockdown and/or circKEAP1-overexpression U2OS and MG63 cell lines. Error bars represent three independent experiments. *, **, *** indicates significant differences compared with the control or indicated group at a p value < 0.05, < 0.01, < 0.001, respectively [file 13046_2024_2971_MOESM8_ESM.jpg]
