# Supplementary material for: A tumor suppressor protein encoded by circKEAP1 inhibits osteosarcoma cell stemness and metastasis by promoting vimentin proteasome degradation and activating anti-tumor immunity
Source: J Exp Clin Cancer Res. 2024 Feb 21;43:52. doi: 10.1186/s13046-024-02971-7 (PMC10880370; doi:10.1186/s13046-024-02971-7)
Supplement: Supplementary file 9 — Supplemental Tables 1 and 2 [file 13046_2024_2971_MOESM9_ESM.zip › Table S1.docx]

| **Gene** | **Forward** | **Reverse** |
| --- | --- | --- |
| CircKEAP1 | GTCATGAACGGTGCTGTCAT | TAAGCAACACCACCACCTCT |
| KEAP1 | CTGGAGGATCATACCAAGCAGG | GGATACCCTCAATGGACACCAC |
| divers | GTCATGAACGGTGCTGTCAT | TAAGCAACACCACCACCTCT |
| cover | GTCATGAACGGTGCTGTCAT | TGGCACTCAGGGACCTCC |
| CXCR5 | CACGTTGCACCTTCTCCCAA | GGAATCCCGCCACATGGTAG |
| CXCL9 | CCAGTAGTGAGAAAGGGTCGC | AGGGCTTGGGGCAAATTGTT |
| CXCL1 | TCCAAAGTGTGAACGTGAAGTC | TGGATTTGTCACTGTTCAGCA |
| SOX2 | CCCACCTACAGCATGTCCTACTC | TGGAGTGGGAGGAAGAGGTAAC |
| NANOG | AAGGTCCCGGTCAAGAAACAG | CTTCTGCGTCACACCATTGC |
| U6 | CTCGCTTCGGCAGCACA | AACGCTTCACGAATTTGCGT |
| OCT4 | GTGTTCAGCCAAAAGACCATCT | GTGTTCAGCCAAAAGACCATCT |
| CXCL10 | GTGGCATTCAAGGAGTACCTC | TGATGGCCTTCGATTCTGGATT |
| CCL5 | CCAGCAGTCGTCTTTGTCAC | CTCTGGGTTGGCACACACTT |
| RIG-1 | TGTGCTCCTACAGGTTGTGGA | CACTGGGATCTGATTCGCAAAA |
| CXCL8 | ACTGAGAGTGATTGAGAGTGGAC | AACCCTCTGCACCCAGTTTTC |
| CCL22 | ATCGCCTACAGACTGCACTC | GACGGTAACGGACGTAATCAC |
| CCL18 | GCCAGGAGTTGTGAGTTTCC | AGGAGGTATAGACGAGGCAG |
| CXCL11 | GACGCTGTCTTTGCATAGGC | GGATTTAGGCATCGTTGTCCTTT |
| IFI16 | AGACTGAAGACTGAACCTGAAGA | GAACCCATTGCGGCAAACATA |
| IFITM1 | CCAAGGTCCACCGTGATTAAC | ACCAGTTCAAGAAGAGGGTGTT |
| IFI35 | AACAAAAGGAGCACACGATCA | CTCCGTTCCTAGTCTTGCCAA |
| IFNB | GTGTCAGAAGCTCCTGTGGC | TCATAGATGGTCAATGCGGC |
| IRF7 | TAACACCTGACCGCCACCTA | GCTGATCTCTCCAAGGAGCC |
| IFI44 | TCAGGCTTTGGTGGGCACTA | TATGTCATCCCTGCACAGGC |
| GAPDH | CATGTTCCAATATGATTCCACC | CACTTGATTTTGGAGGGATCTC |
| METTL3 | ACACCACCTCTCTGATCTGGCC | CTCCTGAGCTGCAAACTTCTGC |
| METTL14 | ATGGATAGCCGCTTGCAGG | ACACGGCACCAATGCTGTCG |
| IGF2BP1 | TTCTTGGTCAAATCCGGCTA | TACTGAGCCAGCAGGCTGTC |
| hsa_circ_0007366 | ACTTGTCCAGCGCCGATG | TCCGTGAATTGCTCCTCTGT |
| hsa_circ_0027234 | GTTTGAAACCCTGCGCACT | GATTGGCAGCAGAGAGATCT |
| hsa_circ_0050851 | CATGGCTTCGAGACTCACAC | TCCTCCTTCACTTGCTCTTGT |
| hsa_circ_0001792 | TGATAGAGGGCAGGAAGGGG | CTCTCAGCTCCACCTCCTC |

**Supplemental Table 1. Primers**
