# Supplementary material for: A tumor suppressor protein encoded by circKEAP1 inhibits osteosarcoma cell stemness and metastasis by promoting vimentin proteasome degradation and activating anti-tumor immunity
Source: J Exp Clin Cancer Res. 2024 Feb 21;43:52. doi: 10.1186/s13046-024-02971-7 (PMC10880370; doi:10.1186/s13046-024-02971-7)
Supplement: Supplementary file 9 — Supplemental Tables 1 and 2 [file 13046_2024_2971_MOESM9_ESM.zip › Table S2.doc]

| **Parameters** |  | **Case**  **n=72** | **Expression of circKEAP1** | | **Result** |
| --- | --- | --- | --- | --- | --- |
|  |  |  | **Low (n=56)** | **High (n=16)** |  |
| ***Age*** | ≥25 | 29 | 22 | 7 |  |
|  | <25 | 43 | 34 | 9 | P=0.779 |
| ***Gender*** | Male | 48 | 38 | 10 |  |
|  | Female | 24 | 18 | 6 | p=0.767 |
| ***Tumor site*** | Femur | 21 | 14 | 7 |  |
|  | Tibia | 3 | 2 | 1 |  |
|  | jawbone | 26 | 21 | 5 |  |
|  | other | 22 | 19 | 3 | p =0.426 |
| ***Invasion*** | No | 63 | 51 | 12 |  |
|  | Yes | 9 | 5 | 4 | p=0.103 |
| ***Lung metastasis*** | No | 63 | 51 | 12 |  |
|  | Yes | 9 | 5 | 4 | p=0.103 |

**Supplemental Table 2. Correlation between circKEAP1 expression and OS patients’ clinical parameters**
